# The store-operated Ca^2+^ entry complex comprises a small cluster of STIM1 associated with one Orai1 channel

**DOI:** 10.1073/pnas.2010789118

**Published:** 2021-03-01

**Authors:** Yihan Shen, Nagendra Babu Thillaiappan, Colin W. Taylor

**Affiliations:** ^a^Department of Pharmacology, University of Cambridge, Cambridge, CB2 1PD, United Kingdom;; ^b^Department of Basic Medical Sciences, College of Medicine, QU Health, Qatar University, Doha, Qatar

**Keywords:** Ca^2+^ signaling, membrane contact site, Orai, STIM, store-operated Ca^2+^ entry

## Abstract

Increases in cytosolic Ca^2+^ concentration regulate diverse cellular activities and are usually evoked by opening of Ca^2+^ channels in intracellular Ca^2+^ stores and the plasma membrane (PM). For the many signals that evoke formation of inositol 1,4,5-trisphosphate (IP_3_), IP_3_ receptors coordinate the contributions of these two Ca^2+^ sources by mediating Ca^2+^ release from the endoplasmic reticulum (ER). Loss of Ca^2+^ from the ER then activates store-operated Ca^2+^ entry (SOCE) by causing dimers of STIM1 to cluster and unfurl cytosolic domains that interact with the PM Ca^2+^ channel, Orai1, causing its pore to open. The relative concentrations of STIM1 and Orai1 are important, but most analyses of their interactions use overexpressed proteins that perturb the stoichiometry. We tagged endogenous STIM1 with EGFP using CRISPR/Cas9. SOCE evoked by loss of ER Ca^2+^ was unaffected by the tag. Step-photobleaching analysis of cells with empty Ca^2+^ stores revealed an average of 14.5 STIM1 molecules within each sub-PM punctum. The fluorescence intensity distributions of immunostained Orai1 puncta were minimally affected by store depletion, and similar for Orai1 colocalized with STIM1 puncta or remote from them. We conclude that each native SOCE complex is likely to include only a few STIM1 dimers associated with a single Orai1 channel. Our results, demonstrating that STIM1 does not assemble clusters of interacting Orai channels, suggest mechanisms for digital regulation of SOCE by local depletion of the ER.

## Significance

Store-operated Ca^2+^ entry (SOCE) links Ca^2+^ release from endoplasmic reticulum (ER) to Ca^2+^ entry across the plasma membrane (PM). SOCE is unusual in requiring interaction between proteins in different membranes. STIM1, when it senses loss of ER Ca^2+^, unfurls domains that interact with Orai1 PM Ca^2+^ channels. The stoichiometry of the SOCE complex is contentious, but it determines the regulation and functional consequences of SOCE. We show that native complexes are likely to comprise a single Orai1 channel and a few STIM1 dimers, too few to cluster Orai1 channels. We suggest that SOCE may be digitally regulated by local ER depletion, and that local SOCE-evoked Ca^2+^ fluxes are small enough to allow substantial intracellular redistribution of Ca^2+^ through ER tunnels.

**Fig. 1.**
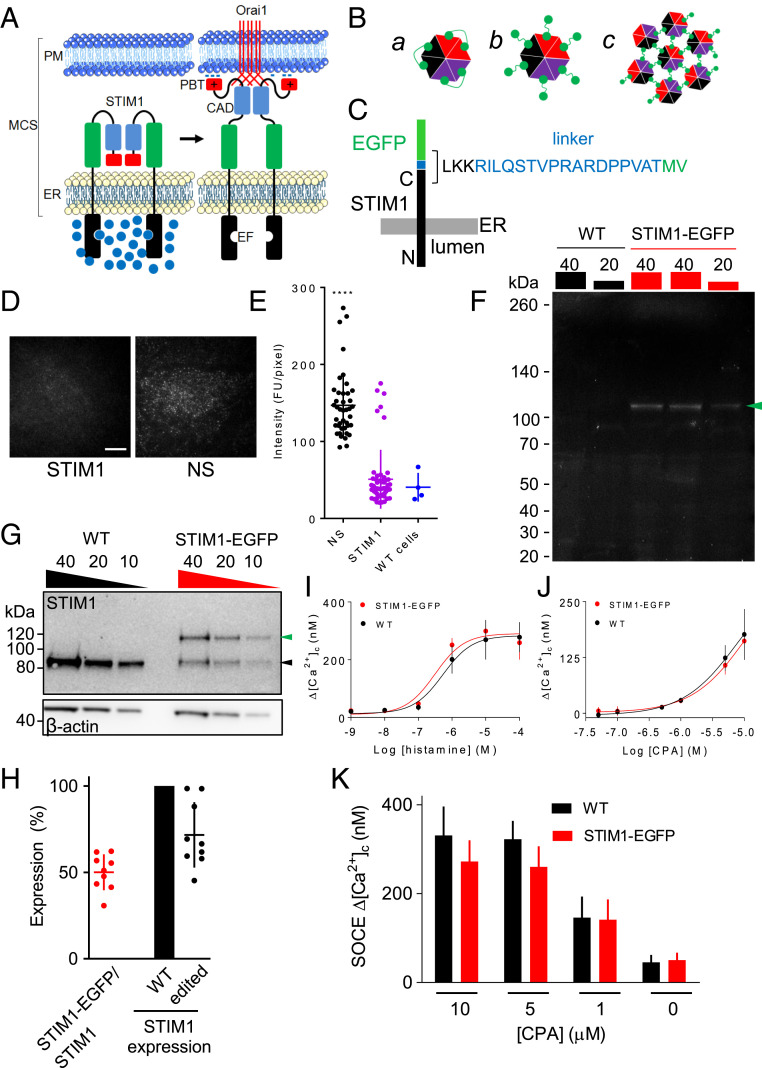
SOCE is unaffected by tagging of endogenous STIM1. (*A*) SOCE is activated when loss of Ca^2+^ from the ER, usually mediated by IP_3_Rs, causes Ca^2+^ to dissociate from the EF hands of dimeric STIM1. This causes STIM1 to unfurl its cytosolic domain, unmasking the C-terminal polybasic tail (PBT) and CRAC (Ca^2+^-release-activated channel)-activation domain (CAD) Association of the PBT with PM phosphoinositides causes STIM1 to accumulate at MCS, where the CAD captures the C-terminal tail of Orai1. Binding of STIM1 to each of the six subunits of Orai1 opens the Ca^2+^ channel, allowing SOCE to occur (9). (*B*) Orai1 is a hexamer, comprising three pairs of dimers (33). Dimeric STIM1 may activate Orai1 by binding as three dimers (*B*, *a*), or as six dimers (*B*, *b*) with the residual STIM1 subunit free to interact with another Orai1 channel (*B*, *c*) (14). (*C*) Structure of the edited STIM1-EGFP. (*D*) TIRF images of STIM1-EGFP HeLa cells treated with STIM1 or nonsilencing (NS) shRNA before emptying of Ca^2+^ stores. (Scale bar, 10 µm.) (*E*) Summary results (individual values, mean ± SD, *n* = 3 independent experiments, each with ∼30 cells analyzed) show whole-cell fluorescence intensities from TIRF images of STIM1-EGFP HeLa cells treated with the indicated shRNA. Results from WT cells are also shown (*n* = 4). ****P < 0.0001, ANOVA with Bonferroni test, relative to WT cells. (*F*) In-gel fluorescence of lysates from WT or STIM1-EGFP HeLa cells (protein loadings in μg). The STIM1-EGFP band (arrow) and molecular mass markers (kDa) are shown. Similar results were obtained in four independent analyses. (*G*) WB for STIM1 and β-actin for WT and STIM1-EGFP HeLa cells. Protein loadings (μg) and molecular mass markers (kDa) are shown. Arrows show positions of native and EGFP-tagged STIM1. (*H*) Summary results (individual values, mean ± SD, *n* = 9) show expression of STIM1-EGFP relative to all STIM1 in STIM1-EGFP HeLa cells (red), and total STIM1 expression in WT and edited cells (black). (*I*) Effects of histamine in Ca^2+^-free HBS on the peak increase in [Ca^2+^]_c_ (Δ[Ca^2+^]_c_) in populations of WT and STIM1-EGFP HeLa cells. Mean ± SEM from four experiments, each with six determinations. (*J*) Effects of CPA in Ca^2+^-free HBS on the peak increase in [Ca^2+^]_c_ (Δ[Ca^2+^]_c_) in populations of WT and STIM1-EGFP HeLa cells. Mean ± SEM from four experiments, each with six determinations. (*K*) Populations of cells were treated (5 min) with CPA in Ca^2+^-free HBS to evoke graded depletion of ER Ca^2+^ stores before addition of extracellular Ca^2+^ (final free [Ca^2+^] ∼10 mM). Results (mean ± SEM, *n* = 6, each with six determinations) show the amplitude of the SOCE in WT and STIM1-EGFP HeLa cells. See also *SI Appendix*, Figs. S1 and S2.

In generating the cytosolic Ca^2+^ signals that regulate cellular activities, cells call upon two sources of Ca^2+^: the extracellular space, accessed through Ca^2+^ channels in the plasma membrane (PM), and Ca^2+^ sequestered within intracellular stores, primarily within the endoplasmic reticulum (ER). In animal cells, the many receptors that stimulate formation of inositol 1,4,5-trisphosphate (IP_3_) provide coordinated access to both Ca^2+^ sources (1). IP_3_ stimulates the opening of IP_3_ receptors (IP_3_R), which are large Ca^2+^-permeable channels expressed mostly within ER membranes. IP_3_ thereby triggers Ca^2+^ release from the ER (2, 3). The link to extracellular Ca^2+^ is provided by store-operated Ca^2+^ entry (SOCE), which is activated by loss of Ca^2+^ from the ER. The reduction in ER free-Ca^2+^ concentration causes Ca^2+^ to dissociate from the luminal Ca^2+^-binding sites of stromal interaction molecule 1 (STIM1), a dimeric protein embedded in ER membranes. This loss of Ca^2+^ causes STIM1 to unfurl cytosolic domains that interact with the PM Ca^2+^ channel, Orai1, causing its pore to open and Ca^2+^ to flow into the cell through the SOCE pathway (Fig. 1*A*) (4, 5). Available evidence suggests that STIM1 must bind to the C-terminal tail of each of the six subunits of an Orai1 channel for optimal activity, with lesser occupancies reducing activity and modifying channel properties (6–10). The interactions between STIM1 and Orai1 occur at membrane contact sites (MCS), where the two membranes are organized to provide a gap of about 10–30 nm, across which the two proteins directly interact (11–13). Orai channels are unusual in having no structural semblance to other ion channels and in having their opening controlled by direct interactions between proteins in different membranes (Fig. 1*A*). Competing models suggest that dimeric STIM1 binds either to a pair of C-terminal tails within a single channel (6 STIM1 molecules per hexameric Orai1 channel) (Fig. 1 *B*, *a*), or that each dimer interacts with only a single C-terminal tail leaving the remaining STIM1 subunit free to cross-link with a different Orai1 channel (12 STIM1 molecules around a single Orai1 channel) (Fig. 1 *B*, *b*) (see references in ref. 14). The latter arrangement has been proposed to allow assembly of close-packed Orai1 clusters (Fig. 1 *B*, *c*) and to explain the variable stoichiometry of Orai1 to STIM1 at MCS (14).

Opening of most ion channels is regulated by changes in membrane potential or by binding of soluble stimuli, where the relationship between stimulus intensity and response is readily amenable to experimental analysis. The unusual behavior of SOCE, where direct interactions between proteins embedded in different membranes control channel opening (Fig. 1*A*), makes it more difficult to define stimulus–response relationships and highlights the need to understand the amounts of STIM1 and Orai1 within the MCS where the interactions occur. When STIM1 or Orai1 are overexpressed their behaviors are perturbed, yet most analyses of their interactions have involved overexpression of the proteins. These difficulties motivated the present study, which was designed to determine the number of native STIM1 molecules associated with each SOCE signaling complex.

## Results

We used CRISPR/Cas9 to attach EGFP to endogenous STIM1 in HeLa cells (Fig. 1*C*) and confirmed the correct attachment of EGFP to the C-terminal of STIM1 by sequencing genomic DNA (*SI Appendix*, Fig. S1 *A*–*C*). In-gel fluorescence, Western blotting with an anti-GFP antibody, and knockdown of STIM1 expression using short hairpin RNA (shRNA) confirmed that STIM1-EGFP was the only EGFP-tagged protein expressed in the STIM1-EGFP HeLa cells (Fig. 1 *D*–*F* and *SI Appendix*, Fig. S1 *D*–*F*). Western blot (WB) analyses revealed that 52 ± 8% of the expressed STIM1 was tagged with EGFP (Fig. 1 *G* and *H*). This is consistent with detection of genomic DNA for both normal and tagged STIM1 in the edited monoclonal STIM1-EGFP HeLa cell line (*SI Appendix*, Fig. S1*C*), and with our HeLa cells having two copies of chromosome 11 on which the *STIM1* gene is located (15). The overall expression of STIM1 was modestly reduced (to 73 ± 18%) in STIM1-EGFP HeLa cells relative to the parental cells (Fig. 1*H*), but there were no significant effects of the editing on Ca^2+^ signaling. Histamine-evoked Ca^2+^ release from the ER, which occurs through IP_3_Rs (15), was indistinguishable in wild-type (WT) and edited cells (Fig. 1*I* and *SI Appendix*, Fig. S2*A*). The Ca^2+^ signals evoked in Ca^2+^-free Hepes-buffered saline (HBS) by cyclopiazonic acid (CPA), which reversibly inhibits the ER Ca^2+^ pump and thereby unmasks an underlying Ca^2+^ leak (*SI Appendix*, Fig. S2*B*), were also unaffected by the gene editing (Fig. 1*J*). This observation, showing the same concentration-dependent loss of ER Ca^2+^ with CPA in the two cell lines, is important because we use CPA to cause graded activation of SOCE. Restoration of extracellular Ca^2+^ to cells treated with various concentrations of CPA in Ca^2+^-free HBS evoked graded increases in cytosolic free Ca^2+^ concentration ([Ca^2+^]_c_), reflecting the activity of SOCE. The amplitude of this SOCE, whether evoked by maximal or submaximal depletion of Ca^2+^ stores, was indistinguishable in the two cell lines (Fig. 1*K* and *SI Appendix*, Fig. S2*B*). The unchanged response to submaximal stimulation of SOCE in the edited cells indicates that STIM1-EGFP is functional. The amplitude of the sustained increase in [Ca^2+^]_c_ evoked by histamine, which probably reports the activity of SOCE, was also indistinguishable in the two cell lines, though smaller than the SOCE evoked by CPA (*SI Appendix*, Fig. S2 *C* and *D*). These results, demonstrating that SOCE is normal in STIM1-EGFP HeLa cells, justify use of these cells for optical analyses of STIM1 redistribution after store depletion.

**Fig. 2.**
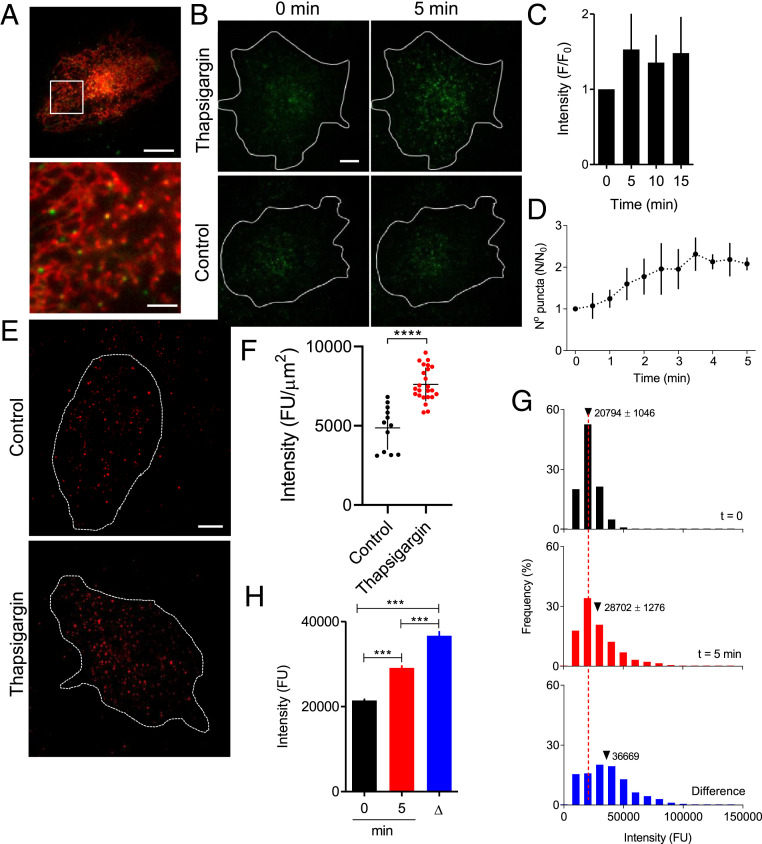
Loss of ER Ca^2+^ causes STIM1 puncta to accumulate beneath the PM. (*A*) TIRF images show distributions of mCherry-ER and STIM1-EGFP in an unstimulated STIM1-EGFP HeLa cell. (Scale bar, 10 μm [2 μm in enlargement of boxed area].) Manders split coefficient for colocalization of STIM1-EGFP with mCh-ER in peripheral regions is 0.87 ± 0.08, mean ± SD, *n* = 9 cells. (*B*) TIRF images of STIM1-EGFP in cells treated with thapsigargin (1 μM, 5 min in Ca^2+^-free HBS) to deplete the ER of Ca^2+^, or in normal HBS without thapsigargin (control). (Scale bar, 10 µm.) (*C*) Summary results (mean ± SD, *n* = 7) show fluorescence (F) recorded from the entire TIRF footprint of each cell at intervals after adding thapsigargin relative to fluorescence immediately before its addition (F_0_). (*D*) Similar analyses of cells treated with CPA (10 μM in Ca^2+^-free HBS) show the number of STIM1-EGFP puncta detected by TIRFM (*SI Appendix*, Fig. S3). Results (N/N_0_, mean ± SD, *n* = 5 cells) show number of puncta at each time (N) relative to number before adding CPA (N_0_). (*E*) WT HeLa cells with replete or empty Ca^2+^ stores (1 µM thapsigargin, 15 min in Ca^2+^-free HBS) were immunostained for STIM1. (Scale bar, 10 µm.) (*F*) Summary results show background-corrected whole-cell TIRF fluorescence from each cell, individual values from 12 (control) and 24 (thapsigargin-treated) cells with mean ± SD ****P < 0.0001, Student’s *t* test. The increase in STIM1 immunofluorescence in WT HeLa cells after depleting Ca^2+^ stores (to 156% of control) is comparable to the increase in EGFP fluorescence in STIM1-EGFP HeLa cells (148%, *C*). (*G*) Fluorescence intensity distributions (*n* = 6 cells) for STIM1 puncta identified by TrackMate in TIRF footprint of entire STIM1-EGFP HeLa cells before or 5 min after adding CPA in Ca^2+^-free HBS. Dashed line shows mean value for cells before stimulation. (*Bottom*) The “difference” distribution: the difference in number of puncta in each fluorescence intensity category for each cell before and after store depletion; the mean fluorescence intensity of these additional puncta is shown. (*H*) Summary results (mean ± 95% CI) show mean fluorescence intensities of STIM1 puncta before and 5 min after CPA addition, and for puncta that appeared as a consequence of store depletion (Δ). See also *SI Appendix*, Fig. S3.

We used total internal reflection fluorescence microscopy (TIRFM) to interrogate cytoplasm immediately beneath the PM and showed that STIM1-EGFP formed puncta within the ER (Fig. 2*A*). Addition of thapsigargin in Ca^2+^-free HBS to irreversibly inhibit the ER Ca^2+^ pump caused accumulation of STIM1-EGFP puncta within the TIRF field. The accumulation was 50% complete by ∼2 min, complete within ∼4 min, and stable thereafter for at least 15 min (Fig. 2 *B*–*D*). The time course and scale of STIM1 accumulation are similar to those from analyses of immunostained native STIM1 (Fig. 2 *E* and *F* and *SI Appendix*, Fig. S2 *E* and *F*) (15), and comparable to analyses using overexpressed STIM1 (16, 17), although the latter vary considerably between studies (*SI Appendix*, Fig. S2*G*). The accumulation of STIM1-EGFP beneath the PM was due to increases in both the number of puncta and their average fluorescence intensity (Fig. 2 *D*, *G*, and *H* and *SI Appendix*, Fig. S3). In step-photobleaching analyses using TIRFM, the amplitude of the final bleaching event was indistinguishable for STIM1-EGFP puncta in stimulated and unstimulated cells (*SI Appendix*, Fig. S4*C*), suggesting that the puncta were all similarly located in the TIRF field. The brighter puncta in cells with empty stores must therefore contain more STIM1-EGFP molecules. These results confirm that loss of Ca^2+^ from the ER causes STIM1-EGFP to form puncta beneath the PM that are both larger and more abundant than in cells with replete Ca^2+^ stores.

**Fig. 3.**
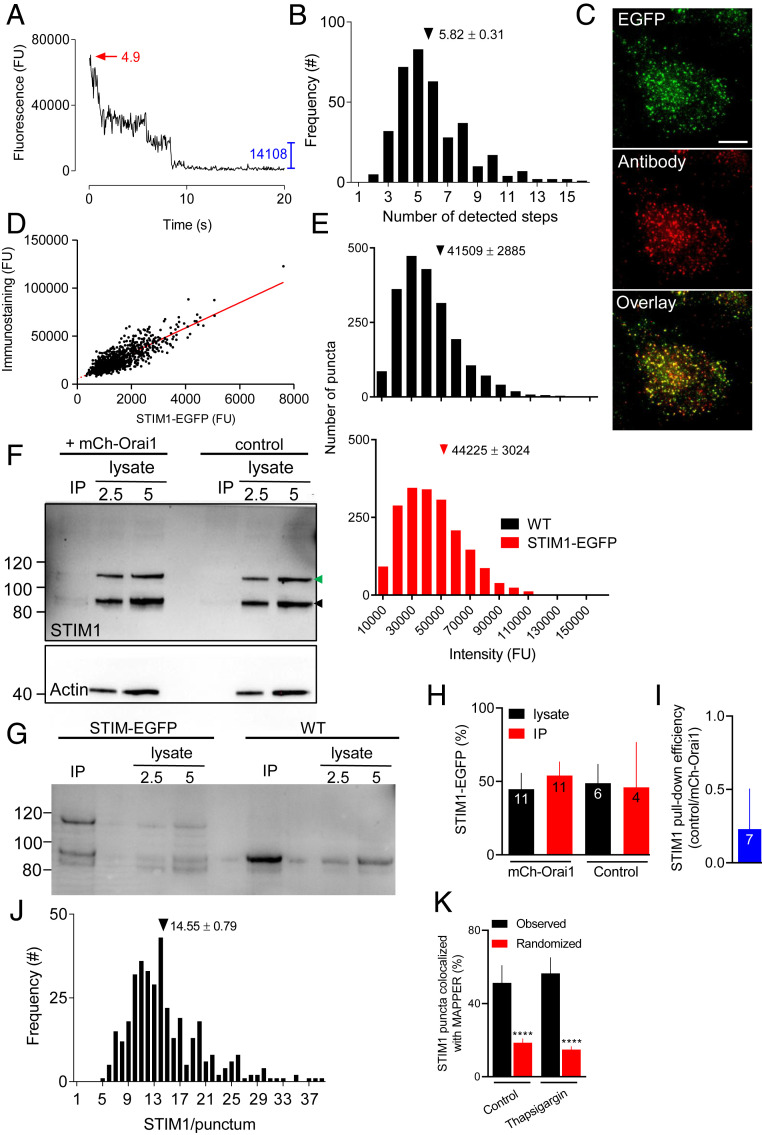
STIM1 and STIM1-EGFP form small puncta after store depletion. (*A*) Typical example of a photobleaching sequence for a STIM1-EGFP punctum in a cell with empty Ca^2+^ stores shows amplitude of the final step (blue) and estimated number of steps (red). Further examples in *SI Appendix*, Fig. S4*B*. (*B*) Frequency distribution for the number of bleaching steps from STIM1-EGFP puncta in cells with empty Ca^2+^ stores. Results are from 365 puncta distributed across 5 cells. Mean number of steps/punctum (± SEM) is shown. (*C*) TIRF images of STIM1-EGFP HeLa cells with empty Ca^2+^ stores immunostained for STIM1 (AbRa594). (Scale bar, 10 µm.) Manders coefficient for overlap of immunostaining with EGFP = 0.72 ± 0.03 (mean ± SD, *n* = 6 cells). (*D*) Summary results (804 puncta from 5 cells, with 37.9 ± 5.0% of cell areas analyzed) show linear relationship between fluorescence from EGFP and immunostaining (least-squares linear correlation coefficient, *r* = 0.834, *P* < 0.0001). (*E*) Frequency distribution of fluorescence intensities of immunostained STIM1 puncta within the entire TIRF footprint in WT (2,116 puncta, 5 cells) and STIM1-EGFP HeLa cells (1,891 puncta, 5 cells). Mean intensities (± SEM) are shown. *P* > 0.05, Student’s *t* test. (*F*) Typical WB for STIM1 (actin in *Lower*) shows immunoreactivity in cell lysates (2.5 and 5 µg protein) and after IP of mCh-Orai1 with RFP-Trap from STIM1-EGFP HeLa cells with or without (control) expression of mCh-Orai1. Cells were treated with thapsigargin (1 µM, 15 min in Ca^2+^-free HBS) before IP. Positions of molecular mass markers (kDa) are shown. Arrows indicate STIM1 and STIM1-EGFP. For cells expressing mCh-Orai1, recoveries (IP/lysate) were 27 ± 18% (mCh-Orai1, mean ± SD, *n* = 8) and 3.6 ± 5.8% (STIM1, *n* = 11). (*G*) Similar IP analysis comparing WT and STIM1-EGFP HeLa cells expressing mCh-Orai1 confirms that the upper band reports STIM1-EGFP. (*H*) Summary shows amount of STIM1-EGFP relative to all STIM1 (%) in lysates and IP for cells with and without mCh-Orai1. Mean ± SD for indicated *n* (values for IP in control cells report only those with detectable STIM1; 4 from 6 WB). (*I*) Although the STIM1-EGFP to all-STIM1 ratio (∼50%) was indistinguishable in control and cells expressing mCh-Orai1, the efficiency of the pull-down of STIM1 was much greater in cells expressing mCh-Orai1. Results show the relative pull-down efficiencies of STIM1 (control/mCh-Orai1 cells) in paired analyses. Mean ± SD, *n* = *7*. The results establish that while the IP does not eliminate nonspecific pull-down of STIM1, the pull-down is significantly greater in cells expressing mCh-Orai1. (*J*) Frequency distribution of the estimated number of STIM1 molecules per punctum in cells with empty Ca^2+^ stores (from *B*). Mean number of STIM1/punctum (± SEM) is shown. (*K*) Colocalization of STIM1-EGFP with mCh-MAPPER puncta (centroid separations < 320 nm) in control and thapsigargin-treated cells. Mean ± SD, *n* = 6 cells; ****P < 0.0001, Student’s *t* test relative to observed. See also *SI Appendix*, Figs. S4–S8.

The fixation required for step-photobleaching analyses has been reported to empty Ca^2+^ stores and activate SOCE (18, 19), but fixation of HeLa cells caused no evident activation of STIM1 (*SI Appendix*, Fig. S5). We also confirmed that the conditions used to identify cells before photobleaching caused negligible prebleaching (*SI Appendix*, Fig. S4*A*). In cells with empty Ca^2+^ stores, the average number of fluorophores per punctum was 5.82 ± 0.31 (Fig. 3 *A* and *B* and *SI Appendix*, Fig. S4 *B* and *C*). Not all puncta resolve the final bleaching step required to estimate the fluorophore content. However, the fluorescence intensity distributions and mean intensities of all puncta and those amenable to step-photobleaching analysis (∼25%, 365/1,477) were indistinguishable (*SI Appendix*, Fig. S4 *D* and *E*). This confirms that our bleaching analysis was unbiased. EGFP tags are detected as fluorescence with ∼80% efficiency (20), suggesting that the average number of STIM1-EGFP molecules in each punctum is 7.28 ± 0.39. A complementary analysis used the fluorescence intensity of STIM1 puncta from cells treated with GFP-siRNA as a calibration signal; it provided a similar estimate of the number of STIM1 molecules in a punctum (*SI Appendix*, Fig. S6).

Since only half the expressed STIM1 is tagged with EGFP, it was important to establish that tagged and native STIM1 coassemble within puncta. Our evidence that SOCE is normal in STIM1-EGFP HeLa cells supports that conclusion (Fig. 1 and *SI Appendix*, Fig. S2), and it is confirmed by additional evidence using STIM1 immunostaining to compare the distribution of all STIM1 with that of STIM1-EGFP. The results demonstrate that puncta identified by the STIM1 antibody, which recognizes native and tagged STIM1 (Fig. 1*G* and *SI Appendix*, Fig. S1*E*), were also labeled with EGFP, and that for each punctum the intensities of EGFP and antibody fluorescence were tightly correlated (Fig. 3 *C* and *D*). The correlation does not alone establish the stoichiometry of STIM1 to STIM1-EGFP, but the consistency of the relationship alongside our demonstration that 50% of STIM1 is tagged (Fig. 1*H*) confirm that tagged and untagged STIM1 mix uniformly in a 1:1 ratio. Furthermore, the fluorescence intensity distributions of STIM1 puncta formed after store depletion and immunostained with STIM1 antibody were indistinguishable in STIM1-EGFP and WT HeLa cells (Fig. 3*E* and *SI Appendix*, Fig. S4*G*). Finally, we expressed mCh-Orai1 in STIM1-EGFP HeLa cells and, although the results were variable, we confirmed that in cells with empty Ca^2+^ stores, STIM1 and STIM1-EGFP were coimmunoprecipitated with mCh-Orai1 and in the same ratio as their expression in the cell lysates (Fig. 3 *F*–*I*). These observations show that the size of the puncta detected after loss of ER Ca^2+^ is unaffected by the EGFP tag, and that untagged STIM1 and STIM1-EGFP mix interchangeably within the puncta that associate with Orai.

We conclude that each punctum in cells with empty Ca^2+^ stores contains an average of 14.5 STIM1 molecules. There is variability between puncta (Fig. 3 *E* and *J*), but the variability is a property of the puncta rather than a limitation of the photobleaching analyses (*SI Appendix*, Fig. S4*H*). In store-depleted cells, most puncta (99.5%) contain at least 6 STIM1 molecules, but only 15.6% contain more than 20 STIM1 and only 2.2% have more than 30 (Fig. 3*J*). These estimates ignore any possible contribution from STIM2, which can oligomerize with STIM1 (21), but in HeLa cells STIM2 expression is only 7.7% that of STIM1 (22). Since ∼50% of STIM1 puncta in cells with empty stores are present before store depletion (Fig. 2*D*), we considered whether this might cause us to substantially underestimate the size of puncta formed as a consequence of ER emptying. By comparing numbers of puncta in each fluorescence intensity category before and after store depletion, we estimated that the mean fluorescence intensity of the STIM1 puncta that appear after store depletion (difference plots in Fig. 2 *G* and *H*) is only 28% greater than that of all puncta in store-depleted cells [mean intensities of 36,669 and 28,702 fluorescence units (FU), respectively]. Including only these “additional” puncta in our analyses would increase our estimate of the number of STIM1 molecules in a punctum in cells with empty Ca^2+^ stores from 14.5 to 18.5 STIM1. Even if we assumed that the brightest 50% of puncta were formed exclusively after store depletion, our estimate of the number of STIM1 molecules per punctum would increase to only 19.0 (Fig. 3*J*). We conclude that in cells with empty stores, native puncta contain rather few STIM1 molecules.

MAPPER is a fluorescent MCS reporter derived from STIM1 (23). We used mCh-MAPPER to identify relationships between STIM1-EGFP and MCS, although we are concerned that MAPPER may perturb accumulation of native STIM1 at MCS, perhaps because STIM1 and MAPPER compete with each other (*SI Appendix*, Fig. S7). Nevertheless, our results suggest a significant colocalization STIM1 puncta with mCh-MAPPER in control and thapsigargin-treated STIM1-EGFP HeLa cells. Under both conditions, ∼50% of STIM1 puncta colocalize with mCh-MAPPER (Fig. 3*K* and *SI Appendix*, Fig. S8*A*). There are, of course, more STIM1 puncta near the PM of cells with empty Ca^2+^ stores (Fig. 2*D*) and so more within identified MCS, but we may underestimate this colocalization if STIM1 and mCh-MAPPER compete for occupancy of MCS (*SI Appendix*, Fig. S7 *H* and *I*).

The mean fluorescence intensity of puncta after store depletion is 138% of that in cells with full Ca^2+^ stores (Fig. 2*G*), suggesting that in unstimulated cells, puncta contain an average of 10.5 STIM1 molecules. This result is similar to the estimate from step-photobleaching analysis of unstimulated cells (8.8 ± 0.5, *SI Appendix*, Fig. S4*F*). We conclude that store depletion causes only a modest (∼50%) increase in the average number of STIM1 molecules within a punctum (from ∼10 to ∼15). The conclusion is substantiated by analyses of WT HeLa cells with immunostained STIM1 puncta, where store depletion caused their average fluorescence intensity to increase by ∼55% (*SI Appendix*, Fig. S4*G*). Hence, most native puncta in cells with empty stores contain enough STIM1 to activate no more than two Orai1 channels if each channel binds three STIM1 dimers (Fig. 1 *B*, *a*), and only one Orai1 channel if each subunit binds its own STIM1 dimer (Fig. 1 *B*, *b*).

We estimated the number of Orai1 Ca^2+^ channels associated with a STIM1 punctum using TIRFM after immunostaining for Orai1 before and after store depletion. We reasoned that the distribution of fluorescence intensities for each immunolabeled spot before store depletion likely reports antibody binding to a single hexameric Orai1 channel, while that determined for Orai1 colocalized with STIM1 after store depletion reports the number of Orai1 channels associated with a SOCE complex. We confirmed both the specificity of the antibody (Ab) by demonstrating loss of immunostaining after treatment with siRNA to Orai1, and the ability of the Ab to resolve increases in the number of Orai1 within an immunolabeled spot (*SI Appendix*, Fig. S9).

**Fig. 4.**
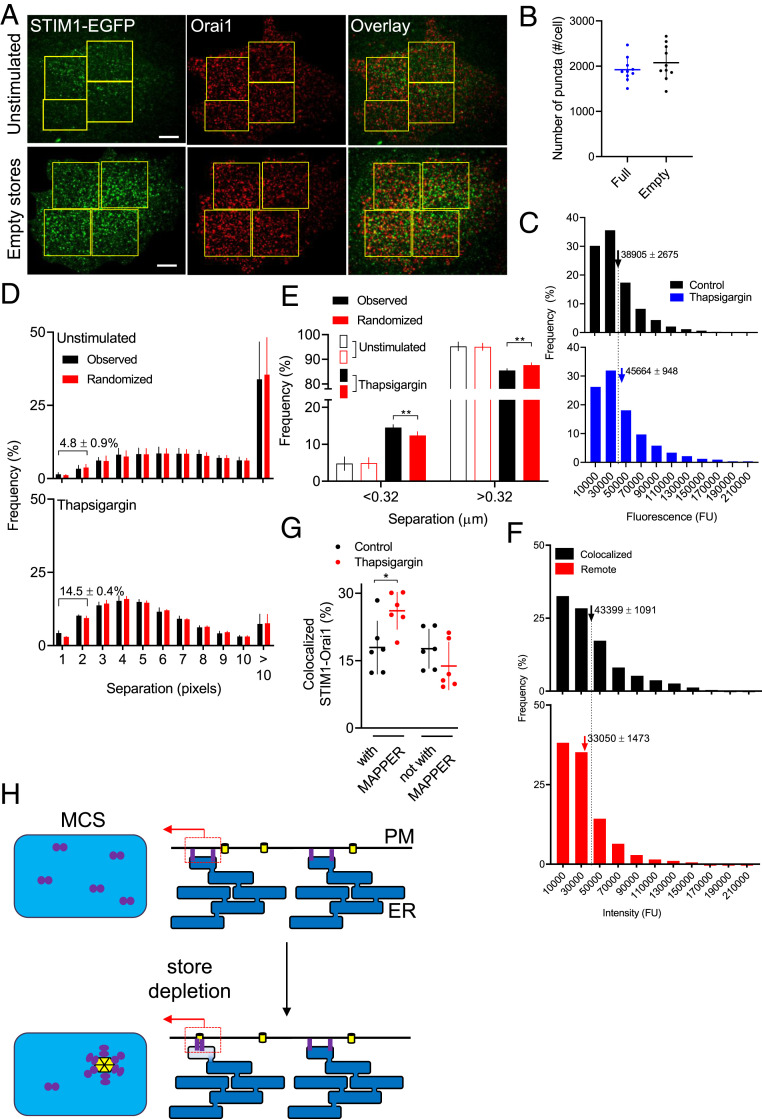
SOCE complex typically includes a few STIM1 dimers and a single Orai1 channel. (*A*) TIRF images show effects of depleting intracellular Ca^2+^ stores (1 µM thapsigargin in Ca^2+^-free HBS, 15 min) on immunostaining with Orai1 Ab (AbRa594 as secondary) in STIM1-EGFP HeLa cells. (Scale bar, 10 µm.) (*B*) Number of Orai1 puncta detected in the TIRF field of STIM1-EGFP HeLa cells before and after store depletion (mean ± SD, *n* = 10). (*C*) Summary results show distributions of fluorescence intensities of immunostained Orai1 puncta in STIM1-EGFP HeLa cells before (10,160 puncta from 5 cells) or after store depletion (10,579 puncta from 5 cells). The *x* axes are truncated for greater clarity; 0.3% of values (included in means) lie beyond the truncation. Mean ± SEM, *P < 0.05, Student’s *t* test. In unstimulated WT cells, the mean fluorescence intensity was 90 ± 12% (*n* = 5 cells) of that in STIM1-EGFP HeLa cells (*n* = 5 cells). (*D*) Distribution of distances between centers of each Orai1 punctum and nearest STIM1 punctum in cells with replete (4,711 Orai1 puncta, 5 cells, each with 3–5 regions of interest [ROI], collectively including ∼55% of cell area) or empty Ca^2+^ stores (6,795 Orai1 puncta, 5 cells). Separations ≤ 320 nm (2 pixels) indicate colocalization. STIM1 puncta were randomly shuffled 100 times within each ROI for comparisons with observed separations (34). (*E*) Frequency distributions segregated into Orai1 colocalized with STIM1 (separation < 0.32 µm, 2 pixels) or not (> 0.32 µm). Mean ± SEM **P < 0.01, *P < 0.05, Student’s *t* test relative to randomized STIM1. (*F*) Distribution of Orai1 fluorescence intensities in STIM1-EGFP HeLa cells with empty Ca^2+^ stores, categorized by whether Orai1 puncta colocalized with STIM1 (separation < 0.32 µm; 1,032 puncta, 5 cells) or were remote from it (> 0.96 µm; 2,334 puncta, 5 cells). Mean ± SEM, *P < 0.05, Student’s *t* test. The *x* axes are truncated for greater clarity; <0.3% of values (included in means) lie beyond the truncation. (*G*) TIRF images of WT HeLa cells expressing GFP-MAPPER and immunonstained for Orai1 and STIM1 were used to identify STIM1 puncta that colocalized with Orai1 (∼40%, *SI Appendix*, Fig. S8 *B*–*D*). Results (mean ± SD) show the distribution of the STIM1 with Orai1 relative to MAPPER for control cells or after treatment with thapsigargin. (*H*) Within each MCS, store depletion modestly increases the number of STIM1 dimers (typically from five to seven) as they assemble around a single Orai1 channel (left). Local redistribution of STIM1 within each tiny MCS (∼300 nm across) (30) may allow each to behave as a digital switch, activated by local depletion of ER Ca^2+^ (*Right*). See also *SI Appendix*, Figs. S7–S10.

The fluorescence intensity distributions for immunostained Orai1 puncta were indistinguishable for WT and STIM1-EGFP HeLa cells, and minimally affected by depletion of the Ca^2+^ stores (Fig. 4 *A*–*C*). We used an object-based colocalization method (24) to measure distances between the centers of each Orai1 punctum and the nearest STIM1 punctum in cells with full and empty stores. The analysis revealed no significant colocalization (centroid separations < 320 nm) in cells with replete Ca^2+^ stores (4.8 ± 0.9%, *n* = 5 cells), but a significant fraction of Orai1 (14.5 ± 0.4%) colocalized with STIM1 in cells with empty stores (Fig. 4 *D* and *E*). In cells with empty stores, there was minimal difference in the intensities of Orai1 puncta associated with STIM1 puncta or remote from them (separations > 960 nm) (Fig. 4*F*). These results confirm that depletion of ER Ca^2+^ stores causes clustering of STIM1 and its association with Orai1, but there is no discernible aggregation of Orai1 channels.

We used GFP-MAPPER to examine relationships between MCS, Orai1, and STIM1 in WT HeLa cells. As with STIM1-EGFP HeLa cells, similar fractions of STIM1 associated with MAPPER in cells with full (39 ± 6%) or empty Ca^2+^ stores (47 ± 3%) (*SI Appendix*, Fig. S8 *B*–*D*). After store depletion, the fraction of STIM1 puncta colocalized with both MAPPER and Orai1 significantly increased (from 17.9 ± 6.0% to 26.1 ± 4.2%, *n* = 6) (Fig. 4*G*). We suggest that the native complex within which STIM1 activates SOCE typically includes only a single Orai1 channel and that most sites where both proteins occur coincide with MCS identified by MAPPER (Fig. 4*H*).

## Discussion

Overexpressed STIM1 perturbs both its interaction with Orai1 (8, 25–28), and the structures of the ER and MCS where SOCE occurs (12, 19). We therefore used cells with one copy of endogenous STIM1 tagged with EGFP (Fig. 1) to define the number of STIM1 molecules within the puncta that regulate Orai1. We confirmed that SOCE was unperturbed in the edited cells (Fig. 1*K*) and that STIM1 and STIM1-EGFP mixed interchangeably (Fig. 3 *C*–*I*). Our results suggest that in cells with empty Ca^2+^ stores, each SOCE complex typically comprises about seven STIM1 dimers and a single Orai1 channel. Whether dimeric STIM1 binds to one or two subunits of a hexameric Orai1 channel is unresolved (Fig. 1*B*). But, with an average of only seven STIM1 dimers in each punctum and only ∼2% of puncta having more than 15 dimers (Fig. 3*J*), there would be no opportunity for the cross-linking of active Orai1 channels by STIM1 reported in cells overexpressing STIM1 (14, 19, 28). Cross-linking might be possible if fewer STIM1 were required to activate Orai1, but the evidence that all six subunits of Orai1 must bind STIM1 for full activation is compelling (9). Furthermore, our direct measurements of Orai1 staining suggest that each SOCE junction typically contains only a single Orai1 channel (Fig. 4 and *SI Appendix*, Fig. S9), consistent with electrophysiological evidence indicating a lack of Ca^2+^-mediated interactions between native Orai1 channels (29). In Jurkat cells, where CRAC (Ca^2+^-release-activated channel) currents are unusually large, comparison of whole-cell SOCE currents with the number of ER-PM MCS suggests about five open Orai1 channels per MCS (30). Hence most mammalian cells, even when intensely stimulated, probably have very few Orai1 channels in each SOCE junction (30).

In HeLa cells, the number of MCS identified by MAPPER in the TIRF field (∼340 per cell, *SI Appendix*, Fig. S7*D*) is comparable to the number of STIM1 puncta in store-depleted cells (∼400/cell), whether identified by immunostaining (Fig. 3*E* and *SI Appendix*, Fig. S4*G*) or as STIM1-EGFP (Fig. 3 *B* and *D* and *SI Appendix*, Figs. S6*B* and S7*G*). There are considerably more Orai1 channels (∼2,000 per cell, Fig. 4*B*) than STIM1 puncta in a cell, and since each STIM1 punctum is unlikely to activate more than a single Orai1 channel (Fig. 4), we suggest that native STIM1 puncta can activate only a small fraction of the Orai1 channels. In cells where STIM1 is more abundant, after overexpression for example, trapping of Orai1 at MCS may be more effective; and if overexpression also allowed assembly of larger STIM1 puncta the efficiency might be further increased by sharing of STIM1 subunits across clustered Orai1 channels (Fig. 1 *B*, *c*).

In the TIRF field of cells with empty Ca^2+^ stores, 26.1 ± 4.2% of STIM1 puncta colocalize with Orai1 at MCS identified by MAPPER (*SI Appendix*, Fig. S8*C*), although as discussed we may underestimate the number of MCS in store-depleted cells (*SI Appendix*, Fig. S7 *H* and *I*). These observations suggest the presence of ∼100 MCS populated by both STIM1 and Orai1 in the TIRF field, suggesting ∼200 active junctions, each with a single active Orai1 channel, in a maximally activated HeLa cell. This is sufficient to account for the increase in [Ca^2+^]_c_ evoked by store depletion (*SI Appendix*, Fig. S10). The modest rate of Ca^2+^ flux into each SOCE junction through a single Orai1 channel (<6,300 Ca^2+^/s) (30) makes it easier to understand how ER Ca^2+^ pumps, with their very low turnover numbers (<40 Ca^2+^/s) (31), may nevertheless sequester appreciable amounts of the incoming Ca^2+^ and redirect it through the ER and IP_3_Rs to distinct intracellular effectors (32).

The increase in size of STIM1 puncta after store depletion is modest, from about five to about seven dimers (Fig. 2*G*), a change that straddles the minimal number of active dimers (six) needed to allow activation of Orai1 in the one-dimer per Orai1 subunit model (Fig. 1 *B*, *b*). We speculate that assembly of native STIM1 puncta around Orai1 after store depletion is driven largely by recruitment of STIM1 dimers already resident within the MCS, allowing each MCS to function as an autonomous digital regulator of SOCE (1, 15) (Fig. 4*H*).

## Materials and Methods

CRISPR/Cas9 was used to attach EGFP to a single copy of the *STIM1* gene in HeLa cells. Appropriate tagging was verified using WB, in-gel fluorescence, and sequencing of genomic DNA. Immunocytochemistry, immunoprecipitation, and measurements of changes in [Ca^2+^]_c_ evoked by SOCE were used to confirm that the EGFP tag did not perturb STIM1 function. TIRFM and step-photobleaching analyses were used to establish numbers of STIM1 molecules within the puncta formed after depletion of intracellular Ca^2+^ stores. Immunostaining with a validated antibody was used to examine the composition of Orai1 puncta. Details of the methods are provided in *SI Appendix*.

## Supplementary Material

Supplementary File

## Data Availability

All study data are included in the article and/or *SI Appendix*.
