# Supplementary material for: The store-operated Ca2+ entry complex comprises a small cluster of STIM1 associated with one Orai1 channel
Source: Proc Natl Acad Sci U S A. 2021 Mar 1;118(10):e2010789118. doi: 10.1073/pnas.2010789118 (PMC7958290; doi:10.1073/pnas.2010789118)
Supplement: Supplementary File [file pnas.2010789118.sapp.pdf]

SUPPORTING INFORMATION

Appendix 1, Materials and Methods

Materials

MATERIAL	SOURCE	IDENTIFIER
**Antibodies**<br>IC, immunocytochemistry; WB, western blot		
Mouse monoclonal anti-β-actin (8H10D10) (WB, 1:20,000)	Cell Signaling Technology, Boston, MA	Cat# 3700;<br>RRID: AB_2243334
Mouse monoclonal anti-mCherry (1C51) (WB: 1:2000)	Abcam, Cambridge, UK	Cat# ab125096;<br>RRID: AB_11133266
Rabbit monoclonal anti-STIM1 (D88E10) (WB, 1:1000; IC, 1:500)	Cell Signaling Technology	Cat# 5668;<br>RRID: AB_10828699
Rat monoclonal anti-GFP (WB, 1:1000)	ChromoTek, Planegg-Martinsreid, Germany	Cat# 3H9-100;<br>RRID: AB_10773374
Goat anti-rat IgG-HRP (WB, 1:5000)	Santa Cruz Biotechnology Inc, Dallas, TX	Cat# sc-2006;<br>RRID: AB_1125219
Mouse monoclonal anti-rabbit IgG-HRP (WB, 1:5000)	Santa Cruz Biotechnology	Cat# sc-2357;<br>RRID: AB_628497
Mouse IgGκ-binding protein-HRP (WB, 1:5000)	Santa Cruz Biotechnology	Cat# sc-516102;<br>RRID: AB_2687626
Rabbit anti-Orai1 (anti-peptide from N-terminal) (WB, 1:1000; IC, 1: 200)	ProSci Inc, Poway, CA	Cat# 4041;<br>RRID: AB_735415
Donkey anti-rabbit IgG-AlexaFluor 594 (AbRa594, IC, 1:500)	ThermoFisher, Waltham, MA	Cat# A21207;<br>RRID: AB_141637
Goat anti-rabbit IgG-AlexaFluor 647 (AbRa647, IC: 1:500)	ThermoFisher	Cat# A21244<br>RRID: AB_2525812
RFP-Trap magnetic agarose	Chromotek	Cat# rtma-20; RRID: AB_2631363
**Bacteria**		
Alpha-select gold efficiency chemically competent cells	Bioline, London, UK	Cat# BIO-85027
**Reagents**		
Agarose	Bioline	Cat# BIO-41025
Anti-Anti (antibiotic-antimycotic)	Sigma-Aldrich, Gillingham, UK	Cat# A5955
BAPTA	Phion Chemicals, Poole, UK	Cat# 81114671
Cell dissociation buffer (enzyme-free)	ThermoFisher	Cat# 13151014
Cyclopiazonic acid (CPA)	Tocris, Abingdon, UK	Cat# 1235
Dimethyl sulfoxide (DMSO)	Sigma-Aldrich	Cat# D2650

DL-dithiothreitol (DTT)	Sigma-Aldrich	Cat# 43816
DNA gel-loading dye	ThermoFisher	Cat# R0611
Dulbecco's Modified Eagle Medium (DMEM)/nutrient mixture F12 with GlutaMAX	ThermoFisher	Cat# 31331
FastDigest NheI	ThermoFisher	Cat# FD0973
FastDigest PvuI	Thermo Fisher	Cat# FD0624
FastDigest XbaI	ThermoFisher	Cat# FD0684
FastDigest buffer	ThermoFisher	Cat# B64
Fibronectin (human)	Millipore (UK) Ltd, Watford, UK	Cat# FC010
Fluo-8 AM	AAT Bioquest, Sunnyvale, CA	Cat# 21080
Foetal bovine serum (FBS)	Sigma-Aldrich	Cat# F7524
GeneRuler 1kb plus DNA ladder	ThermoFisher	Cat# SM1331
Hank's balanced salt solution	ThermoFisher	Cat# 14025
HEPES	Millipore (UK) Ltd	Cat# 391338
Histamine	Sigma-Aldrich	Cat# H7250
Ionomycin	Apollo Scientific, Bredbury, UK	Cat# 56092-81-0
LB agar	Formedium, Hunstanton, UK	Cat# LMM0202
LB broth	Formedium	Cat# LMM0102
Magic Marker XP western protein standards	ThermoFisher	Cat# LC5602
Midori Green advanced DNA stain	Nippon Genetics Europe, Dueren, Germany	Cat# S6-0022
Novex WedgeWell 4-12% Tris-glycine mini gels	ThermoFisher	Cat# XP04125
Novex Tris-glycine SDS running buffer	ThermoFisher	Cat# LC2675
NuPAGE Tris-acetate gels (3-8%)	ThermoFisher	Cat# EA0375
NuPAGE Tris-acetate SDS running buffer	ThermoFisher	Cat# LA0041
NuPAGE LDS sample buffer	ThermoFisher	Cat# NP0007
OptiMEM reduced-serum medium	ThermoFisher	Cat# 31985
Phosphate-buffered saline (PBS)	ThermoFisher	Cat# 10010
cOmplete™, Mini Protease Inhibitor Cocktail	Sigma-Aldrich	Cat# 11836153001
Q5 hot start high-fidelity DNA polymerase	New England Biolabs, Hitchin, UK	Cat# M0493S
siPORT NeoFX transfection reagent	ThermoFisher	Cat# AM4510

Spectra multicolour broad-range protein ladder	ThermoFisher	Cat# 26634
Thapsigargin	Tocris	Cat# 1138
TransIT-LT1 transfection reagent	Mirus, Madison, WI	Cat# MIR 2305
Tris-acetate EDTA buffer	National Diagnostics, Nottingham, UK	Cat# EC-872
TrypLE Express	ThermoFisher	Cat# 12605
siPORT NeoFX transfection agent	ThermoFisher	Cat# AM4510
**Commercial Assays**		
ECL Prime western blotting detection reagent	GE Healthcare, Little Chalfont, UK	Cat# RPN2236
EZ-10 spin column DNA gel extraction kit	Bio Basic, Markham, Ontario, Canada	Cat# BS354
Plasmid maxi kit	QIAGEN, Manchester, UK	Cat# 12165
Quick-DNA miniprep kit	Zymo Research, Tustin, CA	Cat# D3024
**Cell Lines**		
STIM1-EGFP HeLa cells	This study and (1)	**Fig. 1*C* and *SI Appendix,* Fig. S1*A***
HeLa cells	American Type Culture Collection, Teddington, UK	Cat# CCL-2
**Recombinant DNA**		
Donor DNA in pMA-RQ (ampR) vector	ThermoFisher	This study (***SI Appendix,* Fig. S1*A***)
gRNA (sense, caccGGGACA GCTTGTCCTTCCCT; antisense, aaacAGGGAAGGAC AAGCTGTCCC)		This study (***SI Appendix,* Fig. S1*A***)
VP12: human expression plasmid encoding SpCas9-HF1 with NLS and 3xFLAG tag	Addgene (2)	Cat# 72247;<br>RRID: Addgene_72247
BPK1520: human expression plasmid for spCas9 sgRNA	Addgene (2)	Cat# 65777;<br>RRID: Addgene_65777
mCh-Orai1 in pcDNA3.1+		(3)
GFP-MAPPER in pcDNA3.1+		(4)
mCh-MAPPER in pcDNA3.1+		(3)
pSUPER-retro-puro-shSTIM1	Addgene	Cat# 89816;<br>RRID: Addgene_89816
EGFP siRNA	ThermoFisher	Cat # AM4626
Orai1 siRNA1	ThermoFisher	Cat# AM16708, siRNA ID 216908
Orai1 siRNA2	ThermoFisher	Cat# 4392420, siRNA ID s228396

Orai1 siRNA3	ThermoFisher	Cat# 1299001, siRNA ID HSS131373
AllStars Negative Control siRNA	QIAGEN	Cat# 1027281
mCherry-ER-3 plasmid	Addgene	Cat# 55041; RRID: Addgene_55041
PCR Primer 1F: AAATGGTGACTCGGAGCAG	ThermoFisher	This study (***SI Appendix*, Fig. S1*A*)**
PCR Primer 1R: CTTGTGGCCGTTTACGTCG	ThermoFisher	This study (***SI Appendix*, Fig. S1*A*)**
PCR Primer 2F: TTGGCGAGGAAACAGACTCC	ThermoFisher	This study (***SI Appendix*, Fig. S1*A*)**
PCR Primer 2R: GAGCTGAGGGAACAGCAACT	ThermoFisher	This study (***SI Appendix*, Fig. S1*A*)**
**Software**		
MetaMorph, version 7.8.4	Molecular Devices, San Jose, CA	https://www.moleculardevices.com/
Prism 5, version 8	GraphPad, La Jolla, CA	https://www/graphpad.com/
SoftMax Pro, version 7	Molecular Devices	https://www.moleculardevices.com/
TrackMate	(5)	https://imagej.net/TrackMate
Fiji		https://fiji.sc
Fiji Time Series Analyser, version 2.0		https://imagej.nih.gov/ij/plugins/time-series.html
JACoP		https://imagej.nih.gov/ij/plugins/track/jacop.html
DiAna	(6)	https://imagejdocu.tudor.lu/doku.php?id=plugin:analysis:distance_analysis_diana_2d_3d_:start

Cell culture and transient transfections. We used normal HeLa cells and a cell line (STIM1-EGFP HeLa) where one copy of the endogenous *STIM1* gene was modified to cause expression of STIM1 with a C-terminal monomeric EGFP tag (***SI Appendix*, Fig. S1**). HeLa cells are derived from a human cervical cancer.

HeLa cells were grown at 37°C with 5% CO_2 in Dulbecco's Modified Eagle medium/nutrient mixture F12 + GlutaMAX with 10% foetal bovine serum (FBS). Cells were passaged using Gibco TrypLE Express when they were confluent. For imaging, cells were grown on 35-mm imaging dishes (Cellvis, IBL Baustoff+Labor GmbH, Gerasdorfbei, Wein, Austria) coated with fibronectin (10 µg/mL). Regular screening confirmed that cells were

free of mycoplasma. We previously confirmed the authenticity of the HeLa cells using short tandem repeat profiling (3).

For transient transfections (shRNA and protein-encoding plasmids), cells grown to ~70% confluence were transfected with plasmid using TransIT-LT1 according to the manufacturer's instructions (1 µg DNA per 3 µL reagent). Cells were harvested or imaged after 24 hr (8 hr for cells expressing MAPPERs). Transfection with siRNA used cells grown to ~70% confluence and siPORT NeoFX transfection agent according to the manufacturer's instructions. We used 5 nM siRNA (or NS siRNA) for GFP (***SI Appendix*, Fig. S6**) or 150 nM siRNA for Orai (50 nM of each of the three siRNAs, or 150 nM of NS siRNA, ***SI Appendix* Fig. S9 *A* and *B***). Cells were used after 72 hr.

Tagging of endogenous STIM1 with EGFP. We used CRISPR/Cas9 to modify the genome of HeLa cells to allow expression of STIM1 tagged at its C-terminus with monomeric EGFP (7) attached through a short linker (8) (**Fig. 1*C***). The guide RNA (gRNA) was designed to target the 3' UTR (PAM site at position GRCh38:Chr11:4091823, GGG) of the human *STIM1* gene (***SI Appendix*, Fig. S1*A***). DNA encoding the gRNA was inserted into the BPK1520 plasmid at the *BsmB*I cloning site (2). Double-stranded donor DNA was synthesized, and comprised the coding sequence for the linker and monomeric EGFP flanked by sequences (~1000 bp each) complementary to the STIM1 gene on either side of the intended Cas9 cleavage site (***SI Appendix*, Fig. S1*A***). A stretch of 27 base pairs corresponding to the gRNA and PAM at the 3' UTR was omitted from the donor DNA to prevent re-editing. There are no known translational regulatory elements or miRNA target sites within the omitted sequence. Restriction sites for *Nhe*I and *Xba*I were added to the 5′ and 3′ ends of the donor DNA, respectively. Donor DNA was cloned into the pMA-RQ vector, amplified, excised using *Nhe*I and *Xba*I, and purified to provide the linearized donor DNA used for transfection.

For gene-editing, HeLa cells grown to ~60% confluence in a 175-cm^2 flask were co-transfected with VP12 plasmid encoding SpCas9-HF1 (10 µg) (2), BPK1520 plasmid encoding gRNA (10 µg) and double-stranded donor DNA (20 µg) using TransIT-LT1 reagent. Cells were harvested after 48-72 hr, washed in phosphate-buffered saline (PBS: 1.06 mM KH_2PO_4, 155 mM NaCl, 3 mM Na_2HPO_4, pH 7.3) containing FBS (1%), and re-suspended (~10^7 cells/mL) in sorting medium (PBS with 1 mM EDTA, 25 mM HEPES, 1% FBS, pH 7.0). Cells were sorted by fluorescence-activated cell sorting (FACS, excitation 488 nm, emission 525 nm) using a modular flow multilaser sorter flow cytometer (DakoCytomation,

Beckmann Coulter). Cells with the brightest EGFP fluorescence (~0.1% of the sorted population) were collected into growth medium containing Anti-Anti (streptomycin, 100 μg/mL; penicillin, 100 units/mL; and amphotericin B, 250 ng/mL) and FBS (20%). Polyclonal cells were cultured in this medium for two passages, and then in normal growth medium without antibiotics before FACS sorting as single cells into 96-well plates. Cells were then cultured in Anti-Anti-containing medium to select monoclonal cell lines for two passages before transferring to normal growth medium without Anti-Anti. A single monoclonal STIM1-EGFP HeLa cell line (C13) was used for the work reported here (1).

Analysis of genomic DNA. Genomic DNA was extracted using a Quick-DNA mini prep kit. PCR using Q5 hot start high-fidelity DNA polymerase was used to amplify the *EGFP* sequence corresponding to the intron/exon-12 boundary of *STIM1*, linker and *EGFP* within the donor region (primers 1F and 1R), and the *STIM1-EGFP* sequence outside the donor region (primers 2F and 2R) (***SI Appendix*, Fig. S1*A***). The PCR products were purified by agarose gel electrophoresis, extracted using an EZ-10 spin-column DNA gel extraction kit, and sequenced (Source Bioscience, UK).

Western blotting and in-gel fluorescence. Confluent cells in a T75 flask were harvested (~2×10^7 cells/mL) into lysis buffer (150 mM NaCl, 0.5 mM EDTA, 1% Triton X-100, 10 mM Tris, pH 7.5) containing protease inhibitor cocktail, incubated (1 hr, 4°C) and then sonicated (3 × 10 s, Transonic ultrasonic bath). The supernatant was recovered (20, 000 ×*g*, 30 min, 4°C) and used for analysis.

For WB, proteins were separated on NuPAGE Tris-acetate gels (3-8%), and then transferred to a PVDF membrane using an iBlot gel transfer device (ThermoFisher). The membrane was blocked by incubation (1 hr, 20°C) with BSA (5%) in Tris-buffered saline (TBST: 137 mM NaCl, 20 mM Tris, pH 7.6, 0.1% Tween-20), incubated with primary antibody in TBST (16 hr, 4°C), washed (3 × 5 min) and then with HRP-conjugated secondary antibody in TBST (1 hr, 20°C), and washed (3 × 5 min) before detection of HRP using ECL Prime Western blotting detection reagents and a PXi chemiluminescence detection system (Syngene, Cambridge, UK)

For in-gel fluorescence, proteins were separated, without denaturation, using 4-12% Tris-glycine mini gels (125V, 90 min). GFP fluorescence was captured directly from the gel using a PXi chemiluminescence gel-imaging system (excitation 465 nm, emission 525 nm).

Immunoprecipitation. HeLa cells were used for immunoprecipitation (IP) analyses 24 hr after transfection with mCh-Orai1 (19 µg plasmid DNA/T75 flask). RFP-Trap was used to capture the mCherry tag. Cells in a T75 flask were washed twice with PBS, treated with

thapsigargin (1 µM, 15 min in Ca^{2+}-free HBS), centrifuged (650 ×*g*, 2 min), and supernatants from cell lysates (1-2 mL) were then prepared as described for WB samples. Samples (~400 µL, ~1 mg protein) were incubated with washed RFP-Trap agarose beads (150 µL bead slurry) for 1 hr at 4°C with gentle rotation. The beads were recovered magnetically, washed (3-5 × 500 µL) with TBST containing protease inhibitor cocktail, and the beads were then heated (95°C, 10 min) in LDS sample buffer (80 µL) before analysis by WB.

Measurement of $[Ca^{2+}]_c$ in cell populations. HeLa cells grown to confluence in clear-bottomed 96-well plates coated with fibronectin (10 µg/mL) were loaded with Fluo-8 by incubation (60 min, 20°C) with Fluo-8 AM (2 µM) in HBS (135 mM NaCl, 5.9 mM KCl, 1.2 mM $MgCl_2$, 1.5 mM $CaCl_2$, 11.5 mM glucose and 11.6 mM HEPES, pH 7.3). Cells were used after further incubation (30 min, 20°C) in HBS without Fluo-8 AM. A FlexStation 3 microplate reader (Molecular Devices, San Jose, CA), which also allows automated fluid additions, was used to record Fluo-8 fluorescence (excitation 490 nm, emission 525 nm) at 1.4-s intervals from cells in HBS at 20°C. Fluorescence was collected using SoftMax Pro software. Where indicated, BAPTA (final concentration 2.5 mM) was added to reduce the free $[Ca^{2+}]$ of HBS to ~40 nM. Fluorescence (F) was calibrated to cytosolic free $[Ca^{2+}]$ ($[Ca^{2+}]_c$) from: $[Ca^{2+}]_c = K_D(F-F_{min})/(F_{max}-F)$, where K_D is the equilibrium dissociation constant of Fluo-8 for Ca^{2+} (389 nM), F_{max} and F_{min} are the maximal and minimal fluorescence values determined after addition of $CaCl_2$ (10 mM) and Triton (0.1%) in HBS or BAPTA (2.5 mM) and Triton (0.1%) in Ca^{2+}-free HBS, respectively.

Total internal reflection fluorescence microscopy (TIRFM). For all optical analyses, cells were grown to ~70% confluence on 35-mm imaging dishes (Cellvis) coated with fibronectin (10 µg/mL). For TIRFM, STIM1-EGFP HeLa cells were imaged using an iXon Ultra 897 EMCCD camera (Andor, 512 x 512 pixels) and Olympus IX83 microscope equipped with a 100x oil-immersion TIRF objective (numerical aperture, NA = 1.49), a multi-line laser bank (Cairn; 488, 561 and 647 nm) and an iLas2 targeted laser illumination system (Cairn, Faversham, Kent, UK). Excitation light was passed through a quad dichroic beam splitter and the emitted light was passed through appropriate emission filters (Cairn Optospin, peak/bandwidth: 525/50, 630/75 and 700/75 nm). The camera was used in conventional EM amplification mode (EM gain = 300). TIRF images (~100 nm penetration depth) were captured using MetaMorph software. All images were background corrected by subtracting the mean fluorescence intensity of a region outside the cell using ImageJ or MetaMorph (for photobleaching analyses).

Step-photobleaching analyses. For these analyses, STIM1-EGFP HeLa cells with replete Ca^{2+} stores or after depletion of the stores by incubating cells with thapsigargin (1 μM, 15 min in Ca^{2+}-free HBS), were fixed in paraformaldehyde (PFA, 4% in PBS, 15 min) and washed 3 times with PBS. Treatments were performed in the dark to minimize photobleaching prior to imaging. Fixation of HEK cells with PFA in Ca^{2+}-free PBS has been reported to deplete the ER of Ca^{2+} and activate SOCE (9, 10). We confirmed, by analyses of STIM1 distribution in STIM1-EGFP HeLa cells, that our fixation methods did not activate SOCE (***SI Appendix*****, Fig. S5**).

To minimize the risk of pre-bleaching, which would cause an under-estimate of the number of bleaching steps, effective TIRF illumination was rapidly established on a target cell (< 3 s; laser intensity 6%) before switching to the more intense illumination (30%) used for step-photobleaching. During the pre-bleach illumination conditions, STIM1-EGFP puncta bleached with a half-time ($t_{½}$) of 30 ± 6 s (***SI Appendix*****, Fig. S4*A***), indicating that pre-bleaching would cause the initial fluorescence intensity of puncta to be underestimated by < 7%. For the step-photobleaching analyses, images were captured using TIRFM (30% laser intensity; capture interval, 200 ms). After background correction, puncta were manually identified, and fluorescence intensity changes within these regions of interest (ROI) were quantified using Fiji Time Series Analyser, v.2.0. From the fluorescence intensity profile of each ROI, the number of bleaching steps was computed by dividing the initial fluorescence intensity of each ROI by the amplitude of the final bleaching step (**Fig. 3*A*** and ***SI Appendix*****, Fig. S4*B***).

To estimate the number of STIM1 molecules within a punctum (N), we first corrected the number of bleaching steps (S) to the likely number of underlying EGFP molecules by assuming that EGFP tags are detected as fluorescence with ~80% efficiency (11). Only 50% of STIM1 are EGFP-tagged (**Fig. 1*H***), and since STIM1 and STIM1-EGFP mix freely (**Fig. 1*G*, 3 *C-I*** and ***SI Appendix*****, Fig. S4*G***), we assume that STIM1-EGFP reports the presence of 50% of all STIM1 molecules in a punctum ($N = 2S/0.8$).

Immunostaining. Cells were fixed using paraformaldehyde (4% in PBS, 15 min, 20°C), washed (3 × 5 min), permeabilized in PBS containing Triton X-100 (0.1%, 10 min, 20°C), and washed in PBS (3 × 5 min). Cells were then blocked with skimmed milk (5% in PBS, 30 min, 20°C), followed by BSA (5% in PBS, 30 min, 20°C), incubated with primary antibody in PBS (16 hr, 4°C), washed (4 × 5 min), incubated with fluorescent secondary antibody (AbRa594) in PBS (1 hr, 20°C), washed (4 × 5 min) and used for TIRFM. All analyses of Orai1 immunostaining used TIRF images.

Dual immunostaining for Orai1 and STIM1 (**Fig. 4*G*** and ***SI Appendix*, Fig S8 *B*-*D***) required use of primary antibodies that were each raised in rabbits. Cells were first stained for STIM1 with the primary and secondary Ab (AbRa647), washed with PBS (5 × 15 min with gentle agitation), and then stained for Orai1 using a different secondary Ab (AbRa594). We confirmed, by omission of the primary Orai1 Ab, that the washing allowed selective immunostaining of STIM1 and Orai1.

Automated detection of fluorescent puncta. We used two Fiji plugins to identify fluorescent puncta. Most analyses used TrackMate (5) to automatically identify fluorescent puncta in background-corrected TIRF images (***SI Appendix*, Fig. S3 *A* and *B***). TrackMate uses a 'difference of Gaussians' filter after applying a consistent threshold. For analyses that required measurements of distances between puncta (to define colocalization), we used DiAna (6). This algorithm identifies all local intensity maxima and then uses a threshold to select the local maximum for each punctum. Automated detection of puncta using TrackMate and DiAna identified similar number of puncta (***SI Appendix*, Fig. S3*C***). Most analyses of the fluorescence intensities of puncta used TrackMate, but where prior analysis of colocalization was required, we used DiAna (**Fig. 4*F***).

Quantification and statistical analyses. Analyses were performed without blinding or power calculations to predetermine sample sizes. The only exception relates to analyses of STIM 1 puncta in cells expressing mCh-MAPPER (***SI Appendix*, Fig. S7 *E*-*G***), where cells with 'acceptable' expression of MAPPER were identified before observing the STIM1-EGFP puncta. All sample sizes are described in figure legends. We used Student's *t*-test or, for multiple comparisons, ANOVA and *post hoc* tests (details in figure legends); $P < 0.05$ was considered significant. Colocalization studies used JACoP (for ER and STIM colocalization) or DiAna (6) (for STIM and Orai1 colocalization). For the analyses with DiAna, ROIs (3-5/cell, and together including ~55% of the TIRF footprint) were selected to exclude areas sparsely populated by Orai1 and STIM1 (**Fig. 4*A***). This selection criterion is required to allow valid determination of the statistical significance of any colocalization, for which we randomly shuffled the distribution of STIM1 puncta 100 times within each ROI and then re-assessed distances between each Orai1 punctum and its nearest STIM1 punctum.

Supplemental References

1. Yu F, et al. (2019) Remodeling of ER–plasma membrane contact sites but not STIM1 phosphorylation inhibits Ca^{2+} influx in mitosis. *Proc. Natl. Acad. Sci. USA* 116:10392-10401.
2. Kleinstiver BP, et al. (2015) Engineered CRISPR-Cas9 nucleases with altered PAM specificities. *Nature* 523:481-485.
3. Thillaiappan NB, Chavda AP, Tovey SC, Prole DL, Taylor CW (2017) Ca^{2+} signals initiate at immobile IP_3 receptors adjacent to ER-plasma membrane junctions. *Nat. Commun.* 8:1505.
4. Chang CL, et al. (2013) Feedback regulation of receptor-induced Ca^{2+} signaling mediated by E-Syt1 and Nir2 at endoplasmic reticulum-plasma membrane junctions. *Cell Rep.* 5:813-825.
5. Tinevez JY, et al. (2017) TrackMate: An open and extensible platform for single-particle tracking. *Methods* 115:80-90.
6. Gilles JF, Dos Santos M, Boudier T, Bolte S, Heck N (2017) DiAna, an ImageJ tool for object-based 3D co-localization and distance analysis. *Methods* 115:55-64.
7. von Stetten D, Noirclerc-Savoye M, Goedhart J, Gadella TW, Jr., Royant A (2012) Structure of a fluorescent protein from *Aequorea victoria* bearing the obligate-monomer mutation A206K. *Acta Crystallogr Sect F Struct Biol Cryst Commun* 68:878-882.
8. Navarro-Borelly L, et al. (2008) STIM1-Orai1 interactions and Orai1 conformational changes revealed by live-cell FRET microscopy. *J. Physiol.* 586:5383-5401.
9. Demuro A, et al. (2011) Subunit stoichiometry of human Orai1 and Orai3 channels in closed and open states. *Proc. Natl. Acad. Sci. USA* 108:17832-17837.
10. Perni S, Dynes JL, Yeromin AV, Cahalan MD, Franzini-Armstrong C (2015) Nanoscale patterning of STIM1 and Orai1 during store-operated Ca^{2+} entry. *Proc. Natl. Acad. Sci. USA* 112:E5533-E5542.
11. Ulbrich MH, Isacoff EY (2007) Subunit counting in membrane-bound proteins. *Nat. Methods* 4:310-321.
12. Kilch T, et al. (2013) Mutations of the Ca^{2+}-sensing stromal interaction molecule STIM1 regulate Ca^{2+} influx by altered oligomerization of STIM1 and by destabilization of the Ca^{2+} channel Orai1. *J. Biol. Chem.* 288:1653-1664.
13. Smyth JT, Dehaven WI, Bird GS, Putney JW, Jr. (2008) Ca^{2+}-store-dependent and -independent reversal of Stim1 localization and function. *J. Cell Sci.* 121:762-772.

14. Ong HL, et al. (2015) STIM2 enhances receptor-stimulated Ca^{2+} signaling by promoting recruitment of STIM1 to the endoplasmic reticulum-plasma membrane junctions. *Sci. Signal.* 8:ra3.
15. Smyth JT, DeHaven WI, Bird GS, Putney JW, Jr. (2007) Role of the microtubule cytoskeleton in the function of the store-operated Ca^{2+} channel activator STIM1. *J. Cell Sci.* 120:3762-3771.
16. Liou J, et al. (2005) STIM is a Ca^{2+} sensor essential for Ca^{2+}-store-depletion-triggered Ca^{2+} influx. *Curr. Biol.* 15:1235-1241.
17. Sharma S, et al. (2013) An siRNA screen for NFAT activation identifies septins as coordinators of store-operated Ca^{2+} entry. *Nature* 499:238-242.
18. Zhang SL, et al. (2005) STIM1 is a Ca^{2+} sensor that activates CRAC channels and migrates from the Ca^{2+} store to the plasma membrane. *Nature* 437:902-905.
19. Wu MM, Buchanan J, Luik RM, Lewis RS (2006) Ca^{2+} store depletion causes STIM1 to accumulate in ER regions closely associated with the plasma membrane. *J. Cell Biol.* 174:803-813.
20. Srikanth S, et al. (2012) Junctate is a Ca^{2+}-sensing structural component of Orai1 and stromal interaction molecule 1 (STIM1). *Proc. Natl. Acad. Sci. USA* 109:8682-8687.
21. Luik RM, Wang B, Prakriya M, Wu MM, Lewis RS (2008) Oligomerization of STIM1 couples ER calcium depletion to CRAC channel activation. *Nature* 454:538-542.
22. Baba Y, et al. (2006) Coupling of STIM1 to store-operated Ca^{2+} entry through its constitutive and inducible movement in the endoplasmic reticulum. *Proc. Natl. Acad. Sci. USA* 103:16704-16709.
23. Zhou Z, Neher I (1993) Mobile and immobile calcium buffers in bovine adrenal chromaffin cells. *J. Physiol.* 469:245-273.
24. Hogan PG (2015) The STIM1-ORAI1 microdomain. *Cell Calcium* 58:357-367.

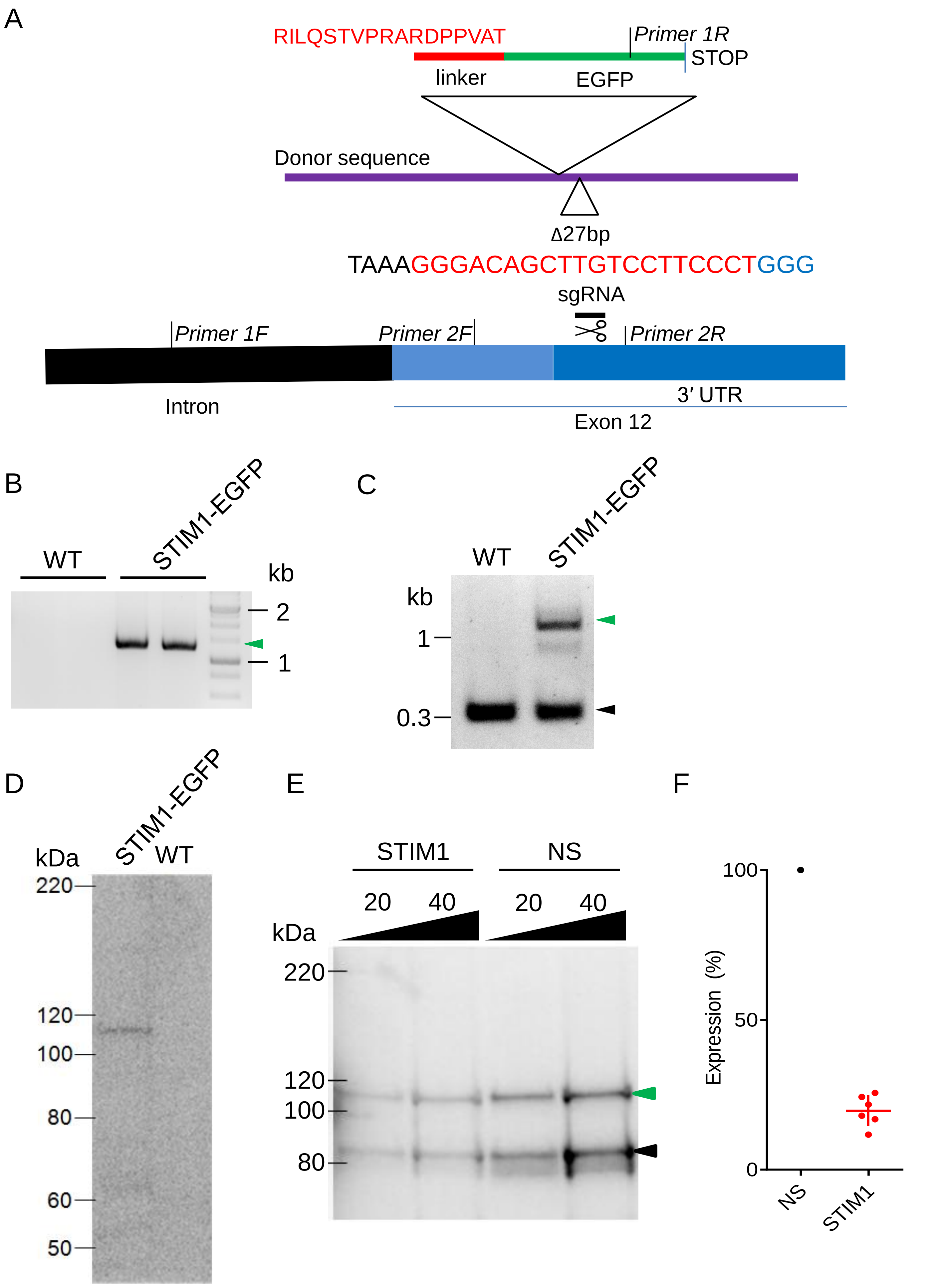

Fig. S1. Tagging of endogenous STIM1 with EGFP using CRISPR/Cas9.
Legend on next page.

Fig.S1. Tagging of endogenous STIM1 with EGFP using CRISPR/Cas9.
Figure on preceding page.

(*A*) CRISPR/Cas9 was used to modify the last coding exon of *STIM1* (exon 12) so as to encode STIM1 with EGFP attached to its C-terminal through a short linker. The donor sequence (comprising 2791 bp with ~1000 bp overlap on either side of the cut site) included a 27-bp deletion to prevent re-editing of the edited gene. The deleted sequence is shown (Δ27bp), with the guide RNA (gRNA) in red letters and the PAM site in blue. The primers (1F, 1R, 2F and 2R) used to confirm correct editing are shown. The peptide sequence of the linker is also shown. (*B*) Genomic DNA from WT and STIM1-EGFP HeLa cells was PCR-amplified using primers 1F and 1R (shown in panel *A*). The band corresponding to STIM1-EGFP (~1.2 kb) is indicated by the green arrow. Two lanes are shown for each isolate. Sequencing confirmed the correct attachment of the linker-EGFP to the C-terminus of STIM1. Locations of calibration markers (kb) are shown. (*C*) Similar analyses of genomic DNA using primers 2F and 2R (shown in panel *A*). A single band (~0.35 kb, black arrow) corresponding to native STIM1 was detected in WT cells. The same band and an additional band (~1 kb, green arrow, corresponding to STIM1 with EGFP attached) were detected in the STIM1-EGFP HeLa cell line. Sequencing of the native band from the edited cell line confirmed that it was identical to that from WT cells. (*D*) WB using an antibody to GFP for lysates (40 µg protein/lane) prepared from STIM1-EGFP or WT HeLa cells. Typical of 4 similar blots. Molecular mass markers (kDa) are shown. (*E*) WB with STIM1 antiserum showing effects of shRNA directed against STIM1 or a non-silencing (NS) shRNA on expression of STIM1 (black arrow) and STIM1-EGFP (green arrow). Molecular mass markers (kDa) are shown. (*F*) Summary (individual values and mean ± SD) show effects of STIM1 shRNA on total expression of STIM1 and STIM1-EGFP relative (%) to expression after treatment with NS shRNA.

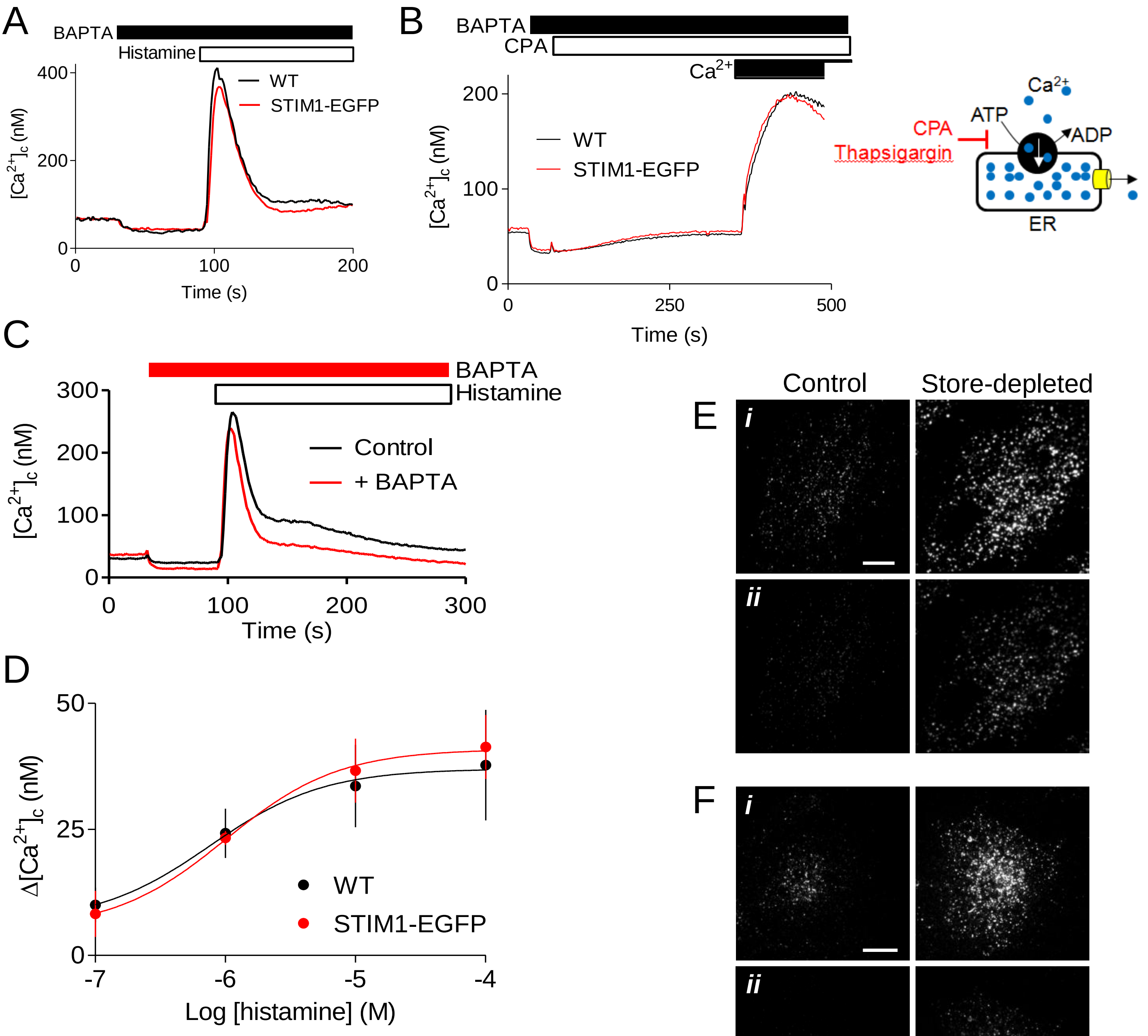

G

Cells	Near-PM STIM1 (%)	Reference
HEK 293	225	Fig. 3A (12)
HEK 293	400	Fig. 1A,B (13)
HEK 293	350	Fig. 3b (14)
HEK 293	350	Fig. 8D (15)
HeLa	240	Fig. 4C (16)
HeLa	350	Fig. 4b (17)
Jurkat	400	Fig. 3g (18)
Jurkat	160	Fig. 4C (19)
Jurkat	215	Fig. 3A (20)
Jurkat	120-250	Fig. 2a,b, S3a,b (21)
DT40	280	Fig. 4D (22)

Fig. S2. Ca^{2+} signals in STIM1-EGFP HeLa cells. Legend on next page.

Fig. S2. Ca^{2+} signals in STIM1-EGFP HeLa cells.
Figure on preceding page.

(A) Populations of Fluo-8-loaded WT or STIM1-EGFP HeLa cells in HBS were treated with BAPTA (2.5 mM) to chelate extracellular Ca^{2+}, and then with histamine (100 µM). Results show means of 6 replicates from one experiment. Summary results in **Fig. 1*I***. (*B*) Populations of Fluo-8-loaded WT or STIM1-EGFP HeLa cells in HBS were treated with BAPTA (2.5 mM) and then with CPA (1 µM). CPA reversibly inhibits the ER Ca^{2+} pump (SERCA), allowing partial depletion of ER Ca^{2+} stores as the basal Ca^{2+} leak is counteracted by diminished SERCA activity (cartoon on right, which shows that both CPA and thapsigargin inhibit SERCA). SOCE was then assessed by restoration of extracellular Ca^{2+} (final free $[Ca^{2+}]$ ~10 mM). Summary results in **Fig. 1*J***. (*C*) Populations of WT cells were stimulated with histamine (100 µM) in HBS (black trace) or HBS after addition of BAPTA (2.5 mM, red trace). Results show means of 6 replicates from one experiment. The difference between the sustained Ca^{2+} signals reflects the contribution from Ca^{2+} entry. (*D*) Summary results (mean ± SEM, *n* = 5, each with 6 determinations) show the amplitude of the Ca^{2+} entry evoked by histamine in WT and STIM1-EGFP HeLa cells (defined as the difference in $[Ca^{2+}]_c$ recorded 210 s after addition of histamine in HBS and Ca^{2+}-free HBS). No significant difference, two-way ANOVA. (*E, F*) Comparison of present and published results on effects of store-depletion on STIM1 accumulation in TIRF field. We have reported immunostaining of STIM1 in HeLa cells (15) similar to the analyses of STIM1-EGFP in **Fig. 2*B***, but shown with different grey-scales. To allow direct comparisons, the results are shown here with comparable grey-scales. The immunostaining results from ref. 15 (Fig. 7a,b) are show with the original display intensities (*Ei*, 500-3000 grey levels) or with a wider range (*Eii*, 500-8000). Images of EGFP-STIM1 from **Fig. 2*B*** are reproduced in grey-scale (*Fi*, 200-1000 grey levels) or with a wider range of levels (*Fii*, 200-3000). The images confirm similar observations using different approaches. Scale bars, 2 µM (*E*) or 5 µM (*F*). (*G*) Summary of published results showing the increase in near-PM STIM1 recorded after depletion of ER Ca^{2+} stores in cells overexpressing STIM1 tagged with a fluorescent protein. Results show fluorescence intensity near the PM as a percentage of that observed without store depletion. The median value is 280% (mean 286 ± 86%). We report values of ~150% for STIM1-EGFP **(Fig. 2*C*)** and 156% for immunostained STIM1 (**Fig. 2F**).

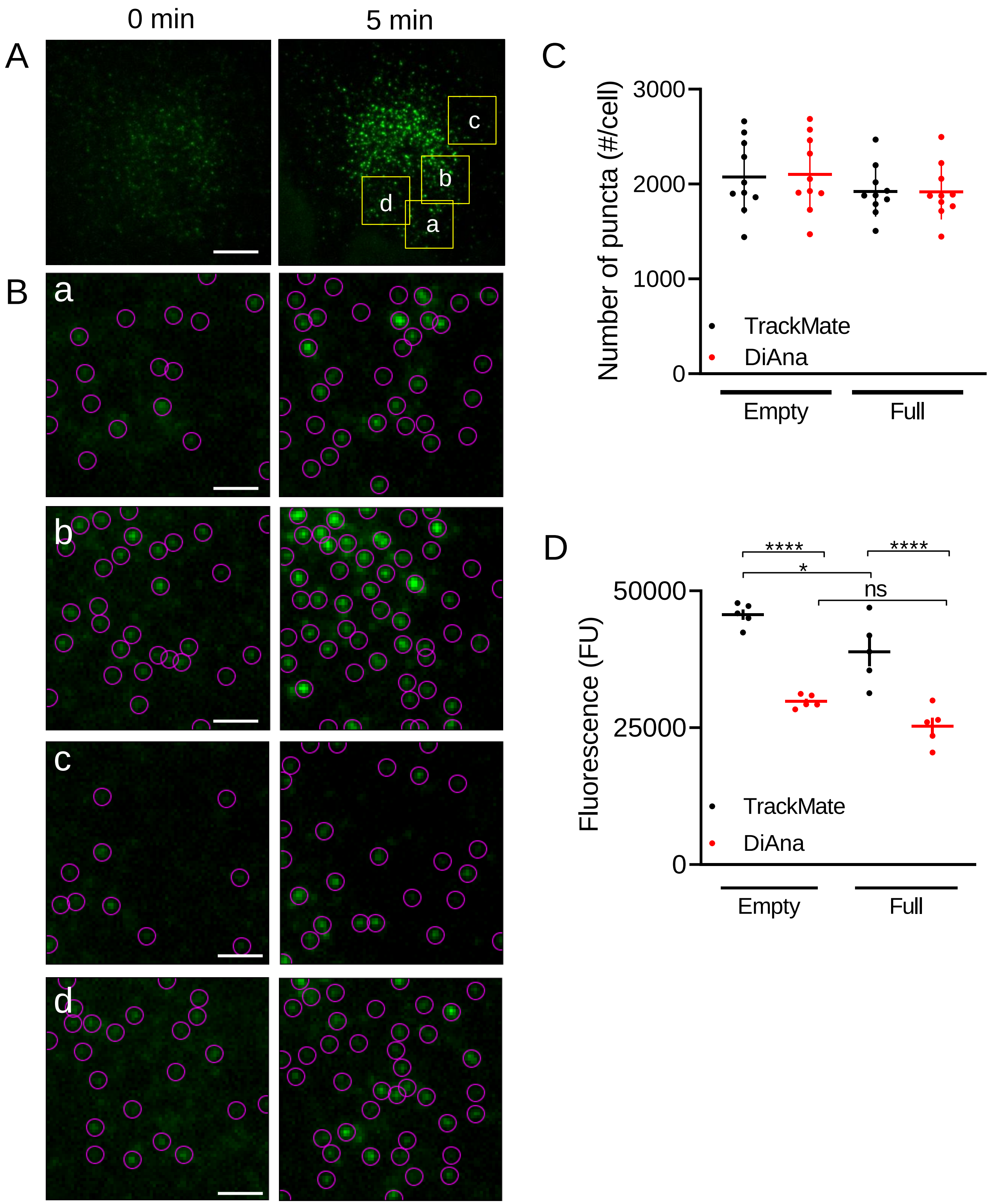

Fig. S3. Automated identification of near-PM STIM1 puncta. (*A*) TIRF images of a STIM1-EGFP HeLa cell before and 5 min after addition of BAPTA (2.5 mM) with CPA (10 µM) to inhibit the ER Ca^{2+} pump. Scale bar, 10 µm. (*B*) Enlargements of boxed areas in panel *A*, with puncta automatically identified using TrackMate shown by circles). Scale bars, 2 µm. Summary results in **Fig. 2, *D, G* and *H***. (*C*) Comparison of TrackMate and DiAna for identification of puncta. STIM1-EGFP HeLa cells with full or empty Ca^{2+} stores were immunostained for Orai1 (primary Ab 1:200), and puncta were identified in the same cells using DiAna or TrackMate. Results show individual values from 10 cells, mean ± SD. No significant difference, one-way ANOVA. (*D*) Similar analysis of the mean fluorescence intensity of the puncta. Results show individual values from 5 cells, mean ± SEM. $^{****}P < 0.0001$, $^{*}P < 0.05$, ns $P > 0.05$, one-way ANOVA with Bonferroni test.

The results establish that DiAna and TrackMate reliably identify similar numbers of puncta, but the fluorescence intensities attributed to puncta differ for the two algorithms.

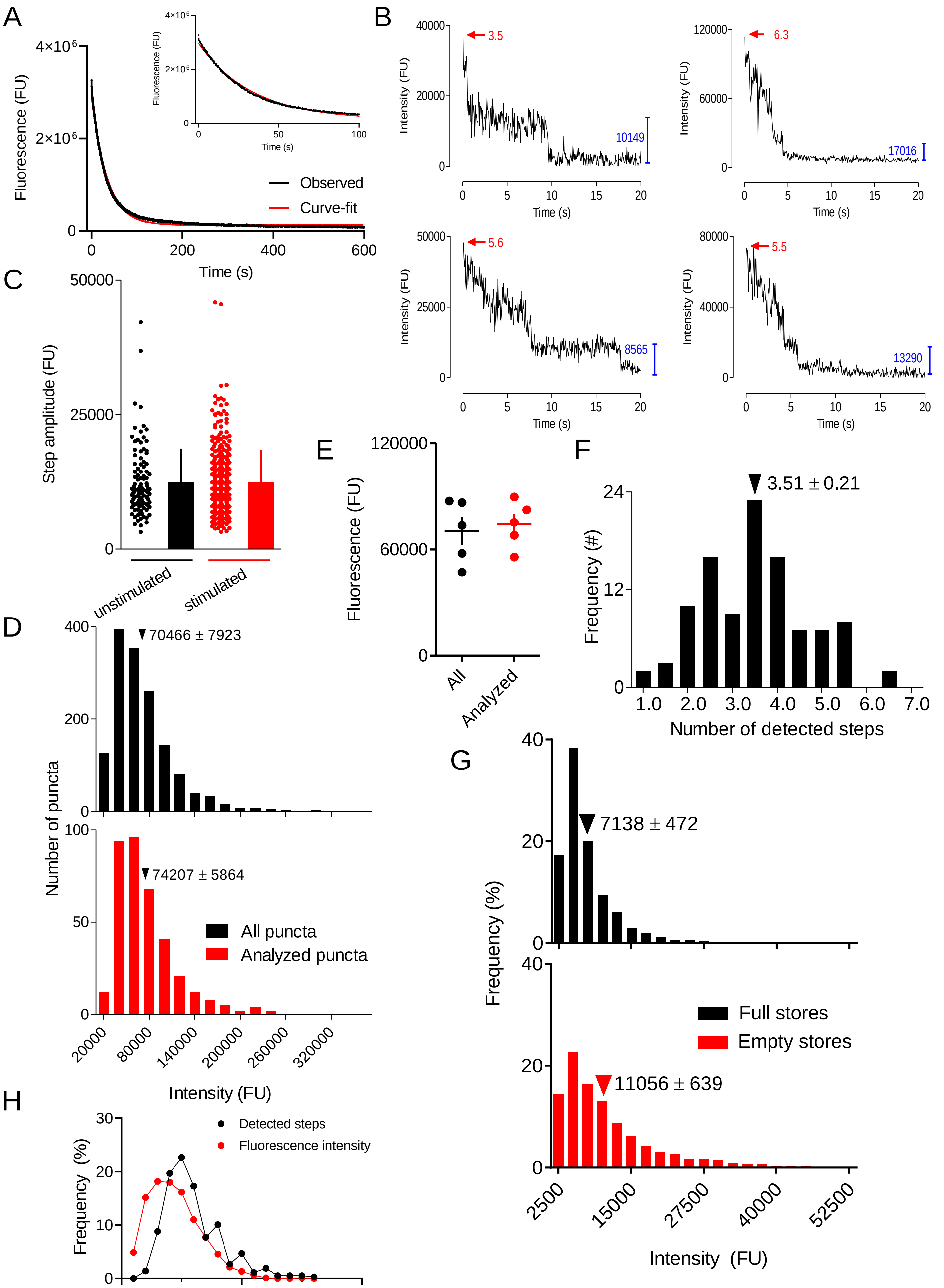

Fig. S4. Photobleaching analyses of STIM1-EGFP puncta.
Legend on next page.

Fig. S4. Photobleaching analyses of STIM1-EGFP puncta**.**
Figure on preceding page.

(*A*) It took <3 s to identify a target cell using low laser power (6%) before initiating photobleaching analysis (30%) (**Fig. 3 *A* and *B***). Fluorescence was recorded from STIM1-EGFP HeLa cells during continuous illumination using the pre-bleach conditions (6% power), and the sum of the fluorescence intensities from all STIM1 puncta within each cell was determined. Results show the time-course of the decay in fluorescence from a single cell (typical of 3 cells), and the fitted mono-exponential from which the half-time ($t_{½}$) was determined. Inset shows the first 100 s enlarged. From 3 independent experiments, $t_{½}$ = 30 ± 6 s. Hence, during the <3 s pre-bleach set-up, we expect to lose no more than 7% of the initial fluorescence of puncta. (*B*) Examples of photobleaching events for STIM1-EGFP puncta recorded using TIRFM of fixed STIM1-EGFP HeLa cells treated with thapsigargin (1 µM) in Ca^{2+}-free HBS. The final event, assumed to report bleaching of a single EGFP, is used to calibrate the initial fluorescence intensity to the number of contributing fluorophores (red in each of the examples). FU, fluorescence unit. (*C*) Summary results show the amplitude of the final bleaching event for STIM1-EGFP puncta in unstimulated cells (107 puncta from 3 cells) and in cells with empty Ca^{2+} stores (365 puncta from 5 cells). Results show individual values and means ± SD, $P > 0.05$, Student's *t*-test. (*D*) Comparison of the fluorescence intensity distributions for STIM1-EGFP puncta amenable to step-photobleaching analysis (i.e. with a clearly resolved final bleaching step; ~25 % of all puncta) and the entire population of puncta. Results are from 5 thapsigargin-treated cells. Fluorescence intensities from step-photobleaching analyses (*C* and *D*) and individual puncta (**Fig. 2 *G* and *H***) are not directly comparable because they use different laser powers. (*E*) Summary results (individual values, mean ± SEM, n = 5 cells) show mean fluorescence intensities of STIM1 puncta, $P > 0.05$, Student's *t*-test. The results (*D* and *E*) demonstrate that the puncta amenable to analysis are an unbiased sample of all STIM1-EGFP puncta. (*F*) Step-photobleaching analysis of STIM1-EGFP in unstimulated STIM1-EGFP HeLa cells. The frequency distribution includes 103 puncta from 3 cells (mean ± SEM). We applied the same assumptions used for analyses of puncta in cells with empty stores (**Fig. 3*J***, and *SI Appendix,* Step-photobleaching analyses) to estimate the number of STIM1 molecules/punctum (*N*) from the number of detected bleaching steps (*S*) in cells with replete Ca^{2+} stores ($N = 2S/0.8$). The results suggest the average number of STIM1 molecules/punctum in unstimulated cells is 8.75 ± 0.54. (*G*) Fluorescence intensity distributions of immunostained STIM1 puncta in WT HeLa cells with full and empty Ca^{2+} stores. Results (6 cells, with 3419 and 3804 puncta analysed for cells with full and empty Ca^{2+} stores, respectively) show mean ± SEM, $^{***}P < 0.001$, Student's *t*-test. Since different laser powers were used for these analyses and those in **Fig. 3*E*** (6% and 10%, respectively), fluorescence intensity values are not directly comparable. (*H*) Frequency distributions of the numbers of detected bleaching steps/punctum for STIM1-EGFP puncta in thapsigargin-treated cells (from **Fig. 3*B***, 365 puncta from 5 cells) and of the fluorescence intensities of the individual puncta (from **Fig 3*E***, 1891 puncta from 5 cells). For each distribution, observations are categorized into 16 bins, and reported as percentages of the entire population. The variability in the step-photobleaching analyses (SD/mean = 0.12, **Fig. 3*B***) and the analyses of fluorescence intensities of puncta (SD/mean = 0.15, **Fig. 3*E***) are similar. Together, these results indicate that the variability in our estimates of the numbers of STIM1 molecules in a punctum reflect biological variability rather than artefacts of our photobleaching analyses.

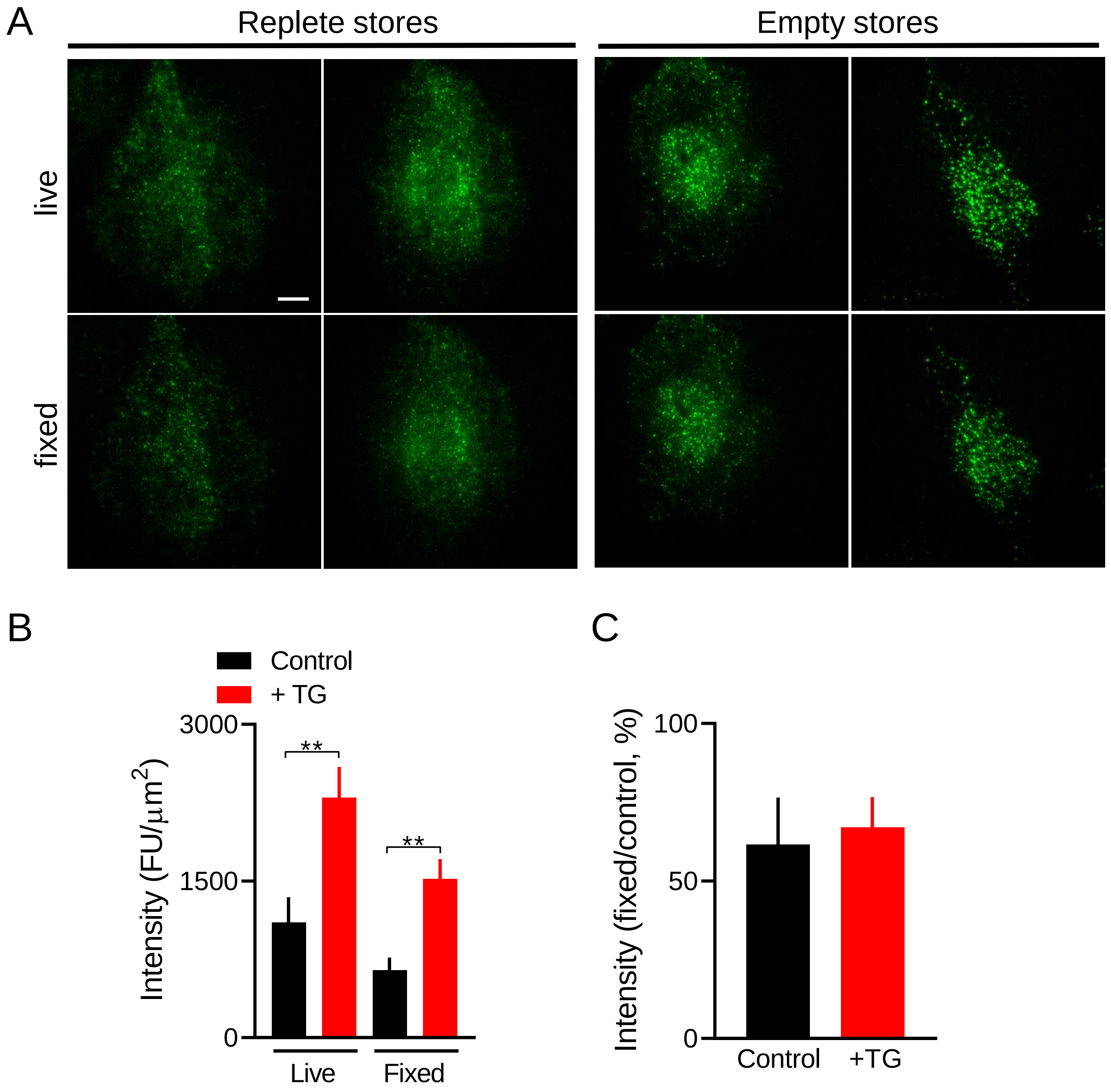

Fig. S5. Fixing cells for immunocytochemical analyses does not activate STIM1. (*A*) TIRF images show STIM1-EGFP HeLa cells before and after on-stage fixation (4% paraformaldehyde, followed by washing), for cells with replete Ca^{2+} stores or after treatment with thapsigargin to empty the stores (TG, 1 µM, 15 min in Ca^{2+}-free HBS). Scale bar, 10 µm. (*B*) Summary results (n = 10 cells for each condition) show the sum of the fluorescence intensities of all STIM1 puncta in each cell (FU/µm^2). Mean ± SEM. $^{**}P < 0.01$, Student's unpaired *t*-test. (*C*) The same observations showing the summed fluorescence intensity of STIM1 puncta for each cell before and after fixation for control cells or cells treated with thapsigargin. No significant difference, Student's unpaired *t*-test.

The results demonstrate that fixation of cells for immunocytochemistry causes some loss of fluorescence, but it does not activate STIM1.

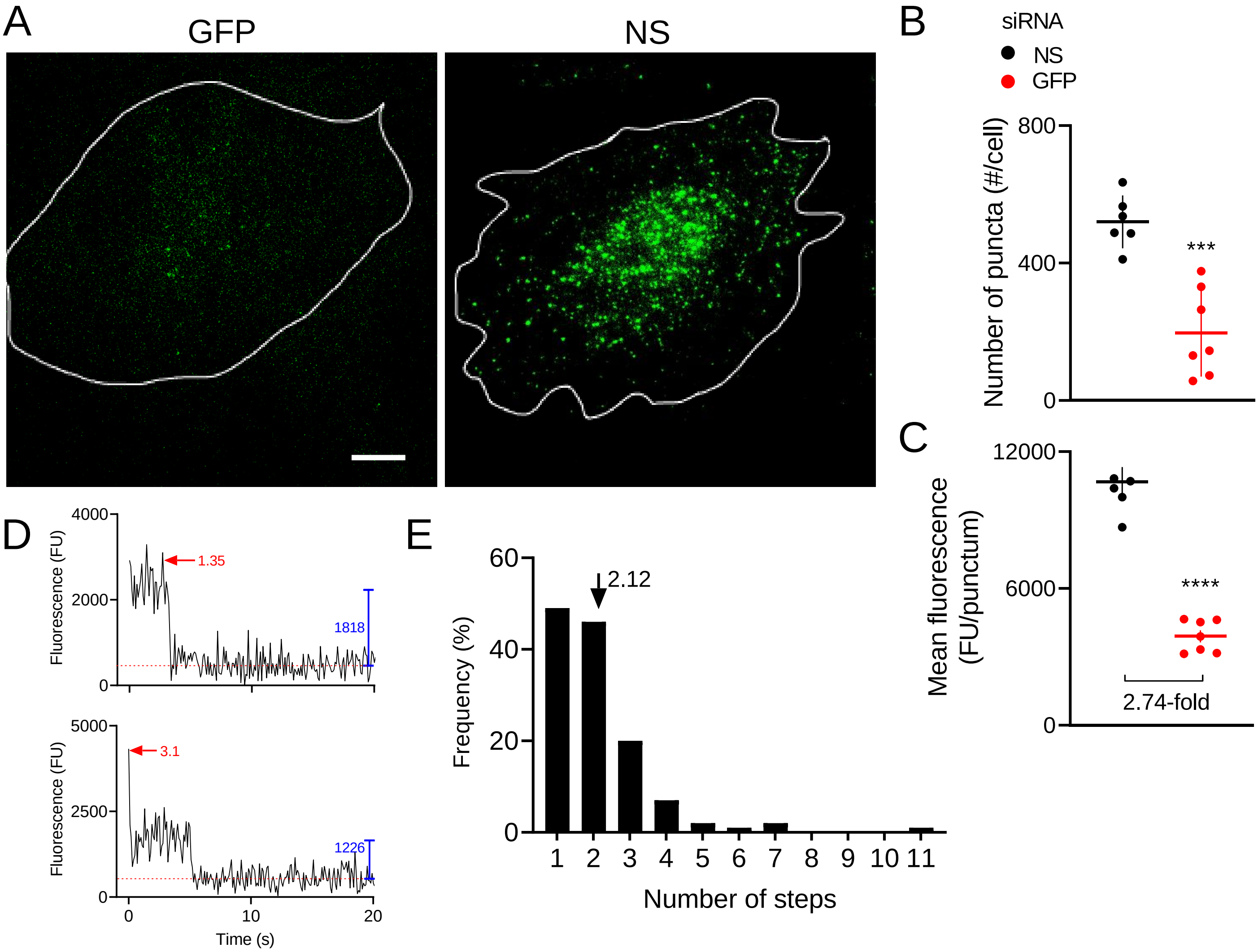

Fig. S6. Estimating the number of STIM1 in a punctum by calibrating against low-level expression of STIM1-EGFP. We used siRNA to GFP to reduce STIM1-EGFP expression in STIM1-EGFP HeLa cells to a level that provided a 'calibration' signal. (*A*) TIRF images of fixed STIM1-EGFP HeLa cells treated with siRNA (72 hr) against GFP or non-silencing (NS) siRNA. Cells were treated with thapsigargin (1 µM, 15 min in Ca^{2+}-free HBS) to empty intracellular Ca^{2+} stores before imaging. A cell in which the GFP-siRNA was effective is shown (typical of those used for the quantitative analysis). Scale bar, 10 µm. (*B*) Numbers of STIM1-EGFP puncta in cells treated with the indicated siRNA. Mean ± SD, n = 6-7 cells. (*C*) Mean fluorescence intensities of the puncta. Mean ± SEM, n = 6-7 cells. $^{****}P < 0.0001$, $^{***}P < 0.001$, Student's unpaired t-test. The code in *B* applies also to panel *C*. The difference in mean intensities for cells treated with NS and GFP siRNA is shown. (*D*) Typical step-photobleaching analysis of STIM1 puncta in cells treated with GFP-siRNA. The initial amplitude (red arrow) and single-step bleaching event (blue) are shown. The latter is smaller here (2272 ± 1302, mean ± SD, n = 128) than in the analyses of native STIM1-EGFP puncta (**Fig. 3E**, ***SI Appendix***, **Fig. S4**) because we used a lower laser intensity for photobleaching (10%). The number of steps was determined by dividing the initial fluorescence amplitude (corrected for residual background fluorescence) by the unitary step amplitude. (*E*) Summary results (128 puncta from 3 cells) show the number of fluorescence steps detected in cells treated with GFP-siRNA. The mean value is shown.

The difference in the mean fluorescence intensity of STIM1 puncta in cells treated with NS or GFP-siRNA (2.74-fold, *C*), and the number of bleaching steps in the puncta from GFP-siRNA-treated cells (2.12, *E*), allows estimation of the fluorophore content of STIM1 puncta in cells treated with NS-siRNA and with empty Ca^{2+} stores (2.74 x 2.12 = 5.81). We assume (***SI Appendix***, **Fig. S4**) that we detect fluorescence from 80% of EGFP molecules and that 50% of STIM1 is tagged. Our analysis, therefore, suggests a mean value of 14.52 STIM1 molecules/punctum in store-depleted cells. This estimate concurs with that derived from conventional step-photobleaching analysis (14.55, **Fig. 3*J***).

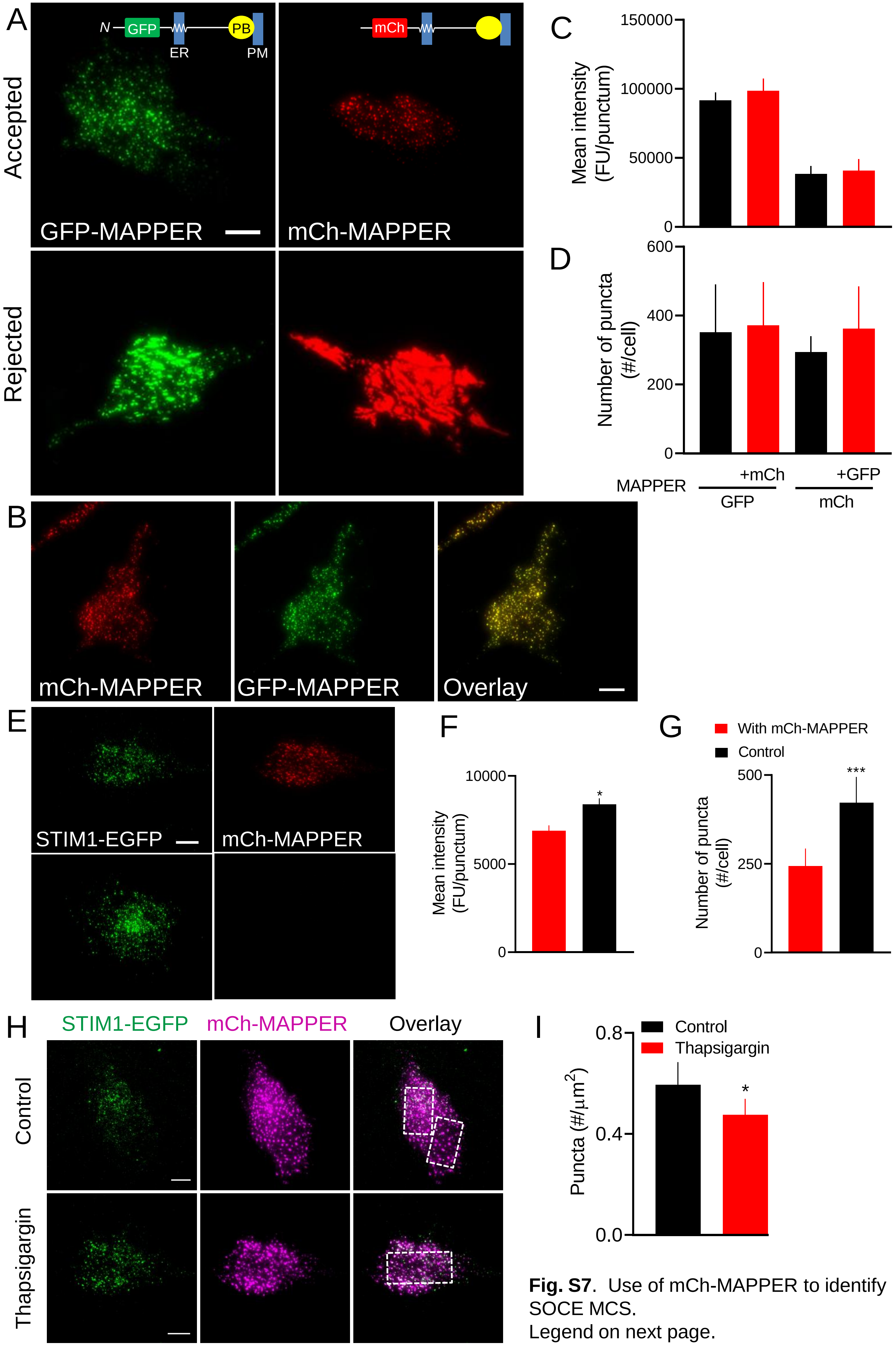

Fig. S7. Use of mCh-MAPPER to identify SOCE MCS.
Legend on next page.

Fig. S7. Use of mCh-MAPPER to identify SOCE MCS.
Figure on preceding page.

MAPPER includes an N-terminal signal sequence that directs it to the ER, the membrane-spanning helix from STIM1, and linkers that allow a polybasic C-terminal tail (PB) to engage PM phosphoinositides (4). GFP-MAPPER has been reported to be a non-perturbing marker of SOCE MCS, at least insofar as it does not affect maximally activated SOCE (4), but we reported that mCh-MAPPER perturbs MCS (3). We therefore compared cells expressing the two MAPPERS.
(*A*) TIRF images of WT HeLa cells expressing GFP-MAPPER or mCh-MAPPER observed 8 hr after transfection; both are shown after depletion of ER Ca^{2+} stores with thapsigargin (1 µM, 15 min in Ca^{2+}-free HBS). Typical images show cells deemed acceptable for analysis (30 from 50 cells for GFP; 29 from 80 cells for mCh) or rejected. Scale bar, 10 µm. (*B*) TIRF images of WT HeLa cells co-expressing GFP-MAPPER and mCh-MAPPER (15 from 44 cells were considered acceptable for analysis). Mander's split coefficients for mCh colocalized with GFP, and for GFP colocalized with mCh were 0.90 ± 0.04 and 0.87 ± 0.04, respectively (mean ± SD, *n* = 5 cells). Scale bar, 10 µm. We note that where expression of MAPPER caused perturbation of MCS ('rejected' cells), there was also substantial colocalization of the two MAPPERS. (*C*) Mean fluorescence intensity in each cell of GFP-MAPPER or mCh-MAPPER puncta (FU/punctum) for HeLa cells expressing one MAPPER or both, recorded 8 hr after transfection. Results are from 5-6 'acceptable' cells (~60% of cells had acceptable expression of MAPPER). Mean ± SEM. No significant differences, Student's unpaired *t*-test. (*D*) Similar analyses of the numbers of GFP-MAPPER or mCh-MAPPER puncta detected in the TIRF field (#/cell). Mean ± SD, *n* = 5-6 cells. No significant differences, one-way ANOVA. Assuming that MAPPER observed in the TIRF field reports ~50% of the MCS in a cell, our analyses suggest ~680 MCS/cell, which is broadly consistent with estimates derived from electron microscopy (~400 /cell, see ref. 30). (*E*) TIRF images of STIM1-EGFP HeLa cells observed 8 hr after transfection with mCh-MAPPER and then treated with thapsigargin (1 µM, 15 min in Ca^{2+}-free HBS) to deplete intracellular stores of Ca^{2+}. Cells were first selected on the basis of 'acceptable' expression of mCh-MAPPER before observing STIM1-EGFP. Representative images show STIM1-EGFP in cells expressing mCh-MAPPER or cells from the same field that appear not to have been transfected. Scale bar, 10 µm. (*F*) Summary results show the average fluorescence intensity of STIM1 puncta, mean ± SEM, *n* = 5 (mCh-MAPPER) or 7 (control) cells. (*G*) Numbers of STIM1 puncta detected in the TIRF field (#/cell), mean ± SD, *n* = 5-7 cells for STIM1-EGFP HeLa cells with or without mCh-MAPPER. $^{*}P < 0.05$, $^{***}P < 0.001$, Student's unpaired *t*-test. (*H*) TIRF images of STIM1-EGFP HeLa cells expressing mCh-MAPPER under control conditions or after depletion of intracellular stores with thapsigrgin (1 µM, 15 min in Ca^{2+}-free HBS). Scale bars, 10 µm. (*I*) Summary results showing numbers of mCh-MAPPER puncta (#/μm^2). Mean ± SD, from 6 cells. $^{*}P$ <0.05, Student's *t*-test. Summary results, from ROI within each cell, reporting colocalization of STIM1 with MAPPER are shown in **Fig. 3*K***.

These results (*A-D*) demonstrate that while expression of mCh-MAPPER does not perturb the reporting of MCS by GFP-MAPPER, mCh-MAPPER reduces the number STIM1 puncta beneath the PM in store-depleted cells (*E-G*), and accumulation of STIM1 beneath the PM in store-depleted cells reduces the number of mCh-MAPPER puncta (*H,I*). Our results are consistent with competition between mCh-MAPPER and STIM1 for occupancy of MCS. We cannot examine the effects of GFP-MAPPER on formation of endogenously tagged STIM1-EGFP puncta, but it seems likely that GFP-MAPPER would also perturb assembly of STIM1 puncta.

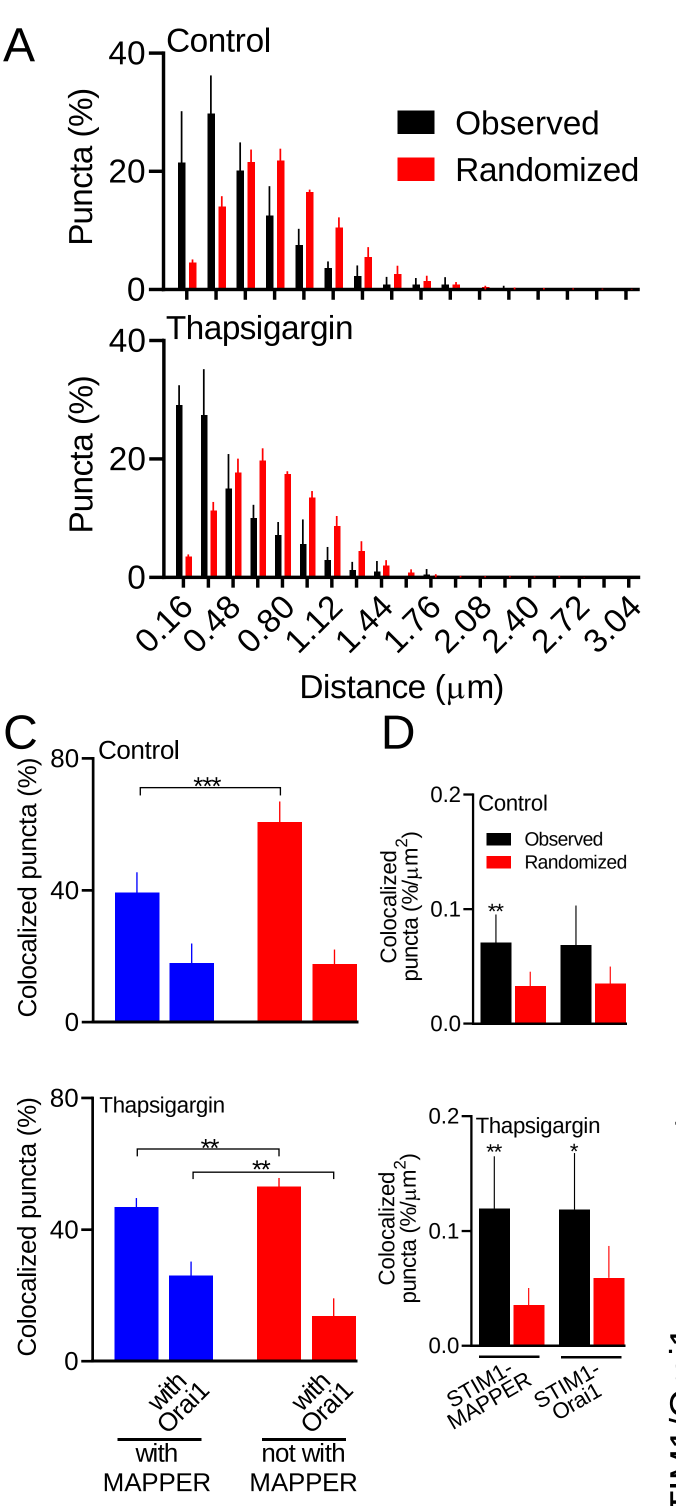

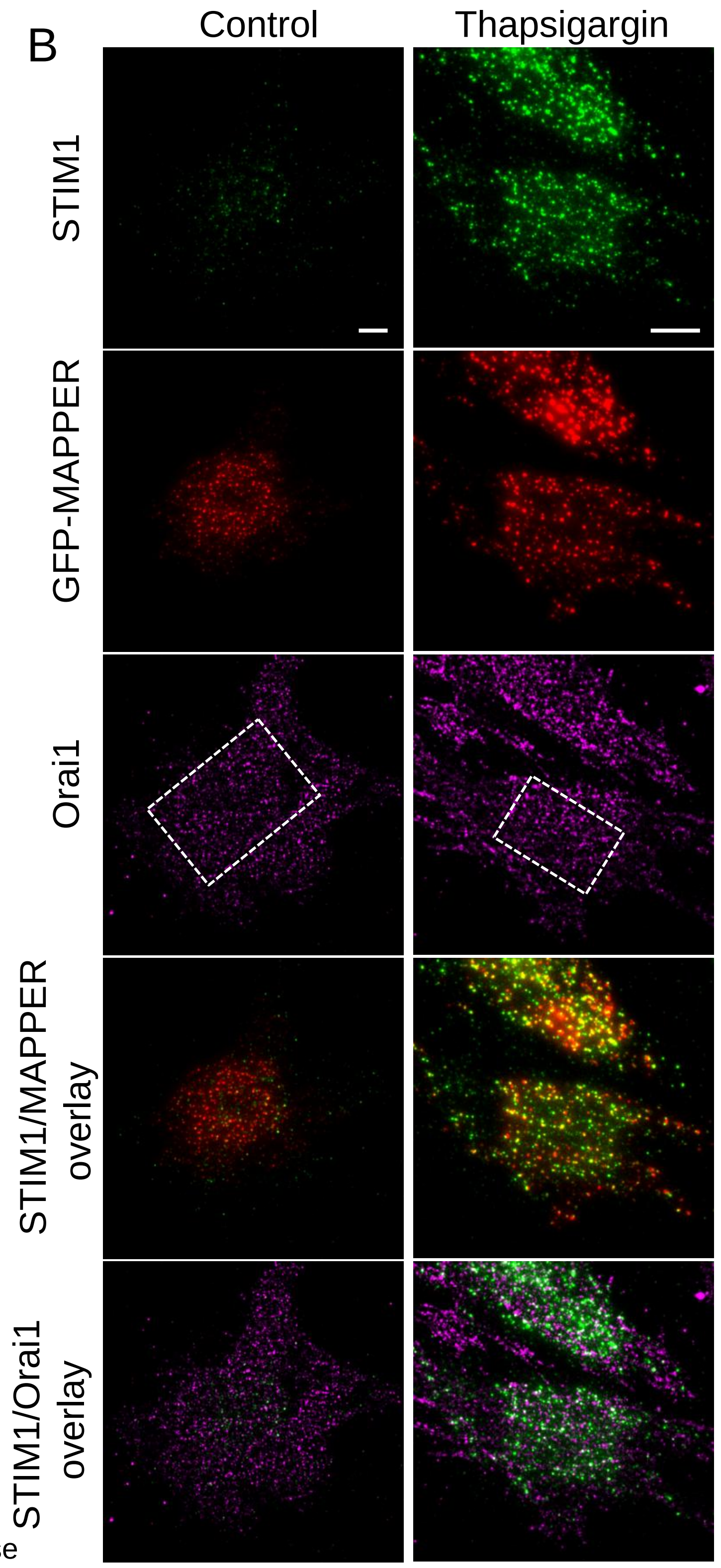

Fig. S8. Relationships between MAPPER, STIM1 and Orai1. (*A*) TIRF images similar to those shown in ***SI Appendix***, **Fig. S7 *E* and *H*** were used to determine distances between each STIM1 punctum and the nearest mCh-MAPPER punctum (centre-to-centre) in STIM1-EGFP HeLa cells with replete or empty Ca^{2+} stores. Results (mean ± SD from 6 cells) show observed separations and separations after 100 random shuffles of mCh-MAPPER puncta. Summary results in **Fig. 3*K***. (*B*) TIRF images of WT HeLa cells expressing GFP-MAPPER and immunostained for Orai1 and STIM1. Scale bars, 10 µm. (*C*) ROI similar to boxed areas in *B* were used to determine numbers of STIM1 puncta colocalized (%, centroid separations < 0.32 µm) with MAPPER and Orai1 in control cells and after treatment with thapsigargin to empty intracellular Ca^{2+} stores. In each case, colocalization was determined by measuring the centre-centre distance from each STIM1 punctum to the nearest MAPPER or Orai1 punctum. Results show all STIM1 puncta colocalized or not with MAPPER, and within these categories the STIM1 puncta that colocalized with Orai1. Mean ± SD from 6 cells. $^{**}P < 0.01$, Student's *t*-test comparing only matched observations that were colocalized or not colocalized with MAPPER. (*D*) Comparison of observed colocalizations with those determined after randomization of the distribution of MAPPER or Orai1 puncta. Mean ± SD from 6 cells. $^{*}P < 0.05$, $^{**}P < 0.01$, Student's *t*-test relative to randomized.

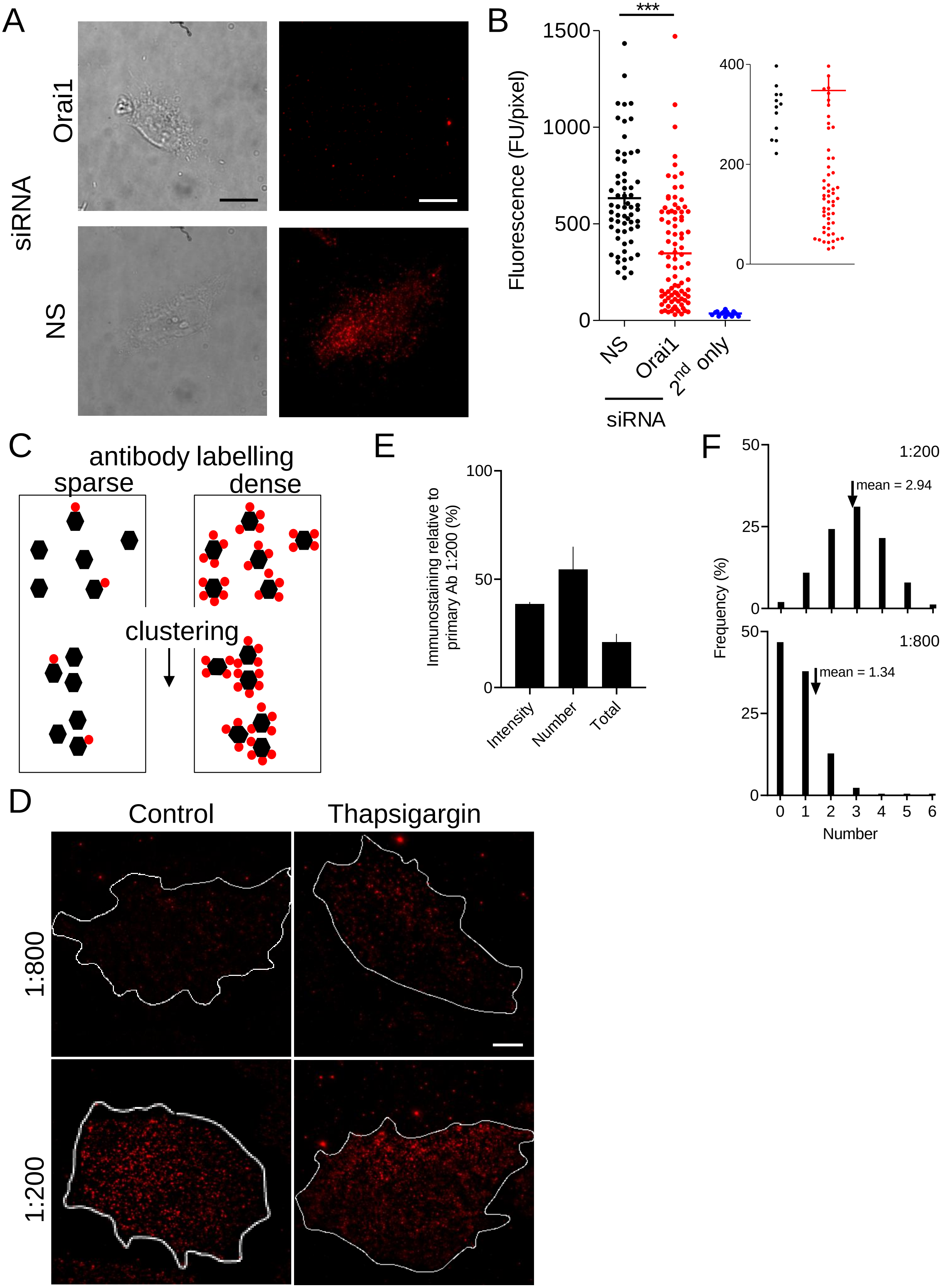

Fig. S9. Validation of analyses of immunostained Orai1 puncta. Legend on next page.

Fig. S9. Validation of analyses of immunostained Orai1 puncta**.**
Figure on preceding page.

(*A*) Brightfield and TIRFM images of unstimulated WT HeLa cells immunostained for Orai1 after treatment with NS or Orai1 siRNA. Scale bar, 10 µm. (*B*) Summary results show mean immunofluorescence for cells treated with NS or Orai1-siRNA or treated with only secondary Ab (individual values, mean ± SD, n = 3 independent siRNA treatments). FU, fluorescence unit. $^{***}P <$ 0.0001, Student's *t*-test. Inset shows lower part of the graph enlarged. These results (*A* and *B*) demonstrate the selectivity of the antibody for immunostaining of Orai1. (*C*) We had to consider whether the Orai1 Ab could detect clustering of channels. If, for example, the Ab (red) only sparsely decorated Orai1 (black), we might fail to detect clustering. We argue that If the usual dilution of primary Ab (1:200) achieves 'dense' labelling, reducing the concentration of primary Ab should reduce the mean fluorescence intensity of immunostained Orai1 puncta. (*D*) TIRF images of unstimulated STIM1-EGFP HeLa cells and after treatment with thapsigargin to empty Ca^{2+} stores, each immunostained for Orai1 with two dilutions of primary Ab. Scale bar,10 µm. (*E*) Summary results for unstimulated cells show mean intensity (FU/punctum) and number (#/cell) of puncta and total TIRF fluorescence for cells immunostained with the Orai1 primary Ab diluted 1:200 or 1:800. Results show values obtained with the 1:800 dilution relative to those with the 1:200 dilution (%), mean ± SEM (intensity) or mean ± SD (number of puncta and total fluorescence), n = 6 cells. Puncta were identified using TrackMate.

We assume that each unstimulated cell has N Orai1 puncta, each with n binding sites for Orai1 Ab; Ab binds independently to each binding site; and n = 6 in an unstimulated cell. A 4-fold dilution of the primary Ab (from 1:200 to 1:800) causes the TIRF immunofluorescence of each cell to decrease to 20.9 ± 3.9% (mean ± SD, n = 6 cells), which is not significantly different from the 25% expected with a linear relationship between Ab concentration and immunostaining. We therefore assume that the probability of Ab binding to a single epitope is P_1 for the 1:800 dilution and $4P_1$ for the 1:200 dilution. From the binomial distribution, the probability ($P_{\text{discovery}}$) of the Ab identifying *any* of the 6 sites within a punctum is: $P_{\text{discovery}} = 1 - (1 - P_1)^6$ for 1:800 and $P_{\text{discovery}} = 1 - (1 - 4P_1)^6$ for 1:200. With the 1:800 dilution, the number of puncta detected is 54.5% of the number detected with the 1:200 Ab dilution (*E*). Hence, $0.545 = P^{1:800}_{\text{discovery}} / P^{1:200}_{\text{discovery}} = (1 - (1 - P_1)^6) / (1 - (1 - 4P_1)^6)$, from which, solving by iteration, $P_1 = 0.119$. (*F*) Predicted frequency of detection of 0-6 subunits of an Orai1 channel with Ab dilutions of 1:200 and 1:800. Predicted mean fluorescence intensity of the detected puncta is shown for each dilution. The predicted values (1:800/1:200 = 46%) aligns with observed values (39%, *E*). With $P_1 = 0.119$, $P_{\text{discovery}}$ for the conditions used for our analyses of fluorescence intensity distributions (primary Ab = 1:200; **Fig. 4**) = $1 - (1 - 4P_1)^6 = 0.98$. We therefore expect to detect most Orai1 puncta ('dense labelling'), with very few 'missed events', and to readily resolve any clustering of Orai1 channels as an increase in the fluorescence intensity of puncta.

Collectively, these results demonstrate the specificity of the Orai1 Ab used (*A,B*) and our ability to resolve clustering of Orai1 channels if it occurred (*C-F*).

A

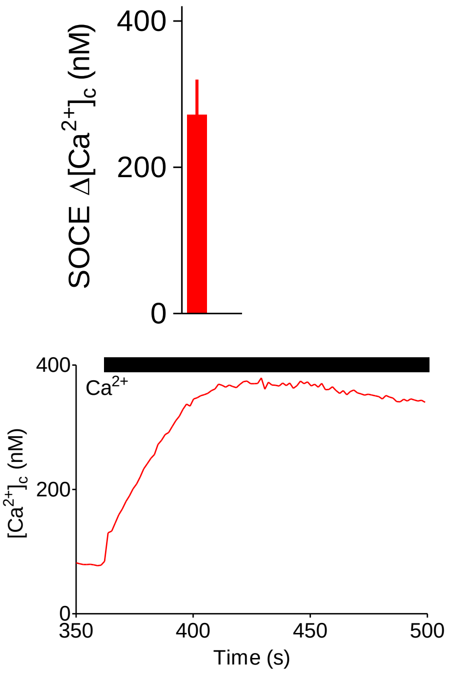

$\Delta[Ca^{2+}]_c$ = 272 ± 48 nM (from **Fig. 1*K***), determined ~60 s after Ca^{2+} restoration to cells treated with 10 μM CPA (from ***SI Appendix,* Fig. S2*B***); half-time ($t_{½}$) is ~15 s). Assuming the cytosolic volume of a HeLa cell (1.6 pL, https://bionumbers.hms.harvard.edu) and a ratio of bound:free Ca^{2+} of ~40 (23), but neglecting any active Ca^{2+} extrusion, this suggests an influx of ~9x10^{-18} mol/cell during the first 15 s.

These estimates suggest a maximal rate of Ca^{2+} influx into each cell of ~350,000 Ca^{2+}/s.

B

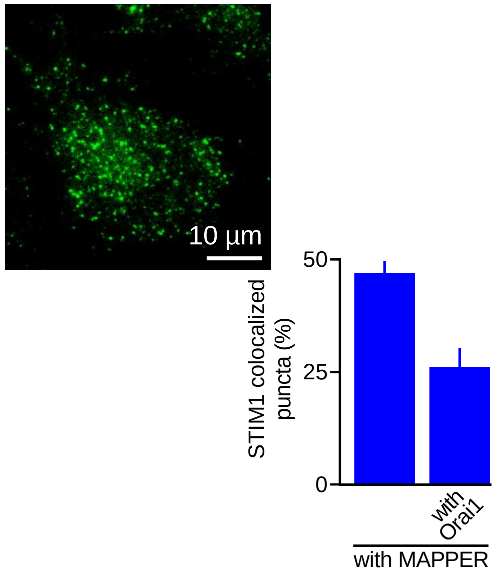

~400 STIM1 puncta in the TIRF field (from **Fig. 3 *B-E*** and ***SI Appendix,* Fig S4*G*, S6*B* and S7*G***).

26% of STIM1 colocalizes with both Orai1 and MAPPER in cells with empty Ca^{2+} stores (***SI Appendix*, Fig. S8*C***), suggesting ~100 STIM1 puncta are present with Orai1 at MCS in the TIRF field.

Assuming TIRF field captures 50% of all PM-associated puncta, we estimate ~200 active Orai1 puncta/cell.

C

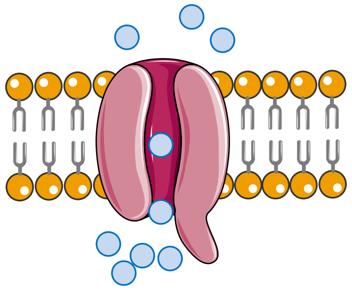

Estimate single active Orai1 conducts 6300 Ca^{2+}/s (24).

Hence, 200 active channels could deliver ~10^6 Ca^{2+}/s into a cell – more than sufficient to account for the observed increase in $[Ca^{2+}]_c$.

Fig. S10. A few active Orai1 channels are sufficient to explain the observed Ca^{2+} signals. The figure illustrates the data and assumptions used to suggest that the relatively few Orai1 channels associated with STIM1 in cells with empty Ca^{2+} stores are sufficient to provide the observed changes in $[Ca^{2+}]_c$ evoked by store depletion.
